# Lateralized Gene Splicing in the Brain is Associated With Lateralized Feeding Behavior in an Asymmetrical Scale-eating Cichlid Fish

**DOI:** 10.1093/gbe/evag237

**Published:** 2026-10-08

**Authors:** Xiaomeng Tian, Axel Meyer

**Affiliations:** Department of Biology, University of Konstanz, Konstanz, Germany; Department of Biology, University of Konstanz, Konstanz, Germany; Museum of Comparative Zoology, Harvard University, Cambridge, MA, USA; CAS Key Laboratory of Tropical Marine Bio-Resources and Ecology, South China Sea Institute of Oceanology, Chinese Academy of Sciences, Guangzhou 510301, China

**Keywords:** left-right asymmetry, brain, alternative splicing, behavioral genetics, cichlids

## Abstract

Lateralized behavior (“handedness”) is a trait observed across all vertebrates, including humans. As an adaptive trait involved in both intraspecific and interspecific interactions, it holds broad evolutionary significance. However, the molecular basis of its brain regulation—particularly post-transcriptional regulation—remains poorly understood. *Perissodus microlepis*, a textbook example in evolutionary biology and an endemic scale-eating cichlid fish from Lake Tanganyika, displays pronounced lateralized feeding behavior that is complemented by its asymmetric head, by which it bites the scales preferentially from only one side or the other side of its prey. Based on data from two previous studies, we investigated the patterns of alternative splicing—an important yet understudied regulatory mechanism—across multiple brain regions in both resting and predatory states of L-eft or the R-ight morphs of this species. We found that the telencephalon, the cognitive center of the vertebrate brain, shows enrichment in functions related to microtubule and neural development in both states. Compared to the resting state, brain regions in the predatory state exhibited more consistent functional profiles, exhibiting pronounced enrichment in the nervous system. We identified several genes whose proportion of alternatively spliced transcripts was strongly correlated with behavioral laterality, establishing a potentially causal link between alternative splicing and handedness. Our findings underscore the significant role of alternative splicing in important adaptive traits, such as lateralized behavior and present a set of candidate genes for further investigation.

Significance Handedness is widespread across vertebrates, including humans, but its molecular basis in the brain remains largely unknown, especially at the level of post-transcriptional regulation. The scale-eating cichlid *Perissodus microlepis*, endemic to Lake Tanganyika, offers a beautiful model for studying behavioral laterality: individuals predominately scrape scales from one side of their prey based on either a left- or right-bent head. By profiling alternative splicing of genes across multiple brain regions in left- versus right-morph fish under resting and predatory conditions, we found that the telencephalon in particular was consistently enriched for alternatively spliced gene with functions related to microtubule organization and neural development, and several genes showed isoform usage strongly associated with behavioral laterality. These findings point toward alternative splicing as a key regulatory layer shaping vertebrate handedness.

## Introduction

Behavioral handedness (or behavioral lateralization) is the systematic, nonrandom preference to use one side of the body over the other. It is widely observed across all vertebrates, such as humans (Coren and Porac 1977), chimpanzees (Lonsdorf and Hopkins 2005), birds (Harris 1989), and reptiles (Danaisawadi et al. 2016). Behavioral handedness has clear evolutionary adaptive significance (Caspar et al. 2022); eg, it can improve the efficiency of information processing (Rogers 2021), enhance foraging and predator-avoidance abilities (Rogers et al. 2004; Ward 2012), and support the evolution of complex cognitive and social behaviors (Frost 1980; Hopkins 2013). In addition, as a behavioral phenotype that helps maintain intraspecific polymorphism (Ghirlanda and Vallortigara 2004; Tian et al. 2025) and contributes to predator–prey interactions (Hulthén et al. 2021), handedness or behavioral lateralization has long been a trait of interest to evolutionary biologists. A well-documented textbook example of behavioral lateralization in fish is *Perissodus microlepis*, a scale-eating cichlid fish endemic to Lake Tanganyika in Africa (Hori 1993; Futuyma 2009). To hunt more efficiently, these fish evolved asymmetrical heads that align with their lateralized foraging behavior (Lee et al. 2010; Stewart and Albertson 2010; Hata et al. 2013; Tian et al. 2024). Left-headed individuals preferentially attack the right side of their prey, while right-headed ones target the left (Lee et al. 2012, 2015; Takeuchi et al. 2012; Raffini et al. 2018a). Despite slight fluctuations, the two morphs have remained at an approximately equal 50:50 ratio in natural populations over time (Hori 1993; Kusche et al. 2012; Lee et al. 2016; Raffini et al. 2018b). This balance is attributed to negative frequency-dependent selection, where the more common morph is at a disadvantage because prey fish that survive these feeding events on their scales—being more frequently attacked from one side—learn to pay better attention to attacks from that side, thereby decreasing the predation success of the more frequent morph (Raffini and Meyer 2018; Berlinghieri et al. 2021). Consequently, *Perissodus microlepis* represents a rare empirical textbook example for negative frequency-dependent selection in evolutionary biology (Futuyma 2009; Raffini and Meyer 2018). The lateralized foraging behavior emerges early, around 2 months of age (A.M., pers. obs.), and persists even in lab-raised fish with no prior hunting experience, indicating a strong innate bias for the behavior (Takeuchi et al. 2022). In support of this hypothesis, a recent genomic study discovered several genome-wide association loci linked to bilaterality, suggesting a polygenic genetic basis for both lateralized behavior and asymmetric head morphology (Tian et al. 2025).

Handedness is not just an asymmetric behavior, but is often also manifested by brain asymmetry (Herve et al. 2006; Sha et al. 2021a). Right-handedness, eg, reflects left-hemisphere dominance (formally called the “dominant temporal lobe”) for controlling the preferred hand, and vice versa (Rogers and Andrew 2002). Genetic research on handedness has been ongoing for decades (Hopkins 2018; Michel et al. 2018; Papadatou-Pastou et al. 2020; Ocklenburg and Gunturkun 2025). Recent large-scale association studies have identified multiple genes linked to handedness, including *MAP2*, *NDRG1*, and *TUBB*, highlighting the role of tubulin genes (Cuellar-Partida et al. 2021; Sha et al. 2021a; Sha et al. 2021b; Schijven et al. 2024; Ocklenburg et al. 2025). Microtubules may contribute to the ontogenesis of handedness during brain development through processes such as axon guidance, axon growth, and the formation of the inner structure of motile cilia (Ocklenburg et al. 2025). Specifically, defective cilia are linked to various syndromes and diseases, known as ciliopathies, which can result in asymmetry defects such as situs inversus (Badano et al. 2006; Fliegauf et al. 2007). The focus on human research has left gaps in our understanding of handedness across species and their evolution. While brain lateralization is linked to behavioral handedness, its molecular basis, especially in nonmammals, remains unclear.

Alternative splicing, a key mechanism of post-transcriptional regulation (Verta and Jacobs 2022; Wright et al. 2022), adds a crucial dimension to understanding the evolutionary mechanisms underlying lateralized behavior. It has gained increasing attention because it enables a single gene to generate multiple protein variants through the selective inclusion or exclusion of specific exons or introns, thereby enhancing proteomic diversity without adding new genes (Jacobs and Elmer 2021; Liu et al. 2024; Tao et al. 2024; Alhabsi et al. 2025). Alternative splicing has been shown to contribute to a wide range of evolutionary innovations (Wright et al. 2022; Hunter and Xing 2026), including increased immune diversity (Schaub and Glasmacher 2017), enhanced phenotypic plasticity (Goeke et al. 2003; Terrapon et al. 2014), hypoxic adaptation (Gothié et al. 2000), and, in particular, the regulation of complex behaviors and neural circuits (Goeke et al. 2003; Hattori et al. 2009). By modulating the splicing of genes expressed in the brain, alternative splicing has been shown to broadly influence animal behavior, such as memory consolidation in honeybees (Ustaoglu et al. 2021), tame versus aggressive behavior in rats (Babenko et al. 2017), aggression (Nakajo et al. 2020), and working-memory learning (Zhong et al. 2023) in zebrafish. The brain is one of the most transcriptionally active tissues, second only to the male testis, with 74% of all proteins being expressed in the human brain (Uhlen et al. 2015). It is also characterized by a higher frequency of alternative splicing events in the brain compared to most other tissues (Yeo et al. 2004; Barbosa-Morais et al. 2012; Merkin et al. 2012). Importantly, beyond exhibiting the highest number of alternative splicing events, the brain also harbors alternative splicing events that are more evolutionarily conserved than those detected in other tissues (Barbosa-Morais et al. 2012; Merkin et al. 2012), suggesting they generally serve conserved functions and their underlying mechanisms may have broad relevance across taxa. Given the role of alternative splicing in expanding protein diversity and its pronounced conservation of brain alternative splicing programs, we hypothesize that this molecular mechanism plays a crucial role in complex behaviors, such as behavioral lateralization.

In this study, we examined whether lateralized behavior is linked to asymmetric gene splicing in the brain using the textbook example of the asymmetric scale-eating cichlid fish *Perissodus microlepis*. To achieve this, we re-analyzed the brain transcriptomes of fish with quantified laterality in resting (Takeuchi et al. 2018) and predatory (Lee et al. 2017) states, using a recently published high-quality reference genome (Tian et al. 2025). Our focus was on the previously unexplored associations between alternative splicing and lateralized behavior across two physiological states, enabled by the high resolution afforded by the new reference genome. First, we examined the expression patterns of both alternative splicing and differential expression across multiple brain regions. Next, we evaluated splicing differences across different brain regions under both physiological conditions. Finally, we incorporated behavioral data to explore the relationship between alternative splicing and lateralized behavior. Specifically, for the fish examined under the predatory state, we further assessed the correlation between splicing variation and the degree of lateralization, identifying genes associated with quantified lateralization levels. We believe that the results extend our understanding of the relationship between laterality and brain lateralization at the molecular level and lay the ground for future research on behavioral genomics.

## Materials and Methods

### Sample Description

We used RNA-seq data from two published studies (Lee et al. 2017; Takeuchi et al. 2018) of brain tissue from both L- and R-morphs of *P. microlepis*: one from fish that had been kept calm and isolated from other fish beforehand (NCBI Bioproject PRJDB6174; hereafter referred to as “the resting state”) and one from fish immediately after a feeding attack (NCBI Bioproject PRJNA353066; hereafter referred to as “the predatory state”).

For the fish collected in the resting state (Takeuchi et al. 2018), behavior laterality was determined based on the widely accepted correlation between behavior laterality and dimorphic mouth asymmetry. The left-right difference in mouth morphology closely corresponds to the preferred attack orientation (Lee et al. 2012), where a left-headed fish preferentially attacks the right side of the prey, and vice versa. The fish showed no intense movements before sampling to make sure the isolated RNA from the fish was in a stationary state. The telencephalon, optic tectum, and hindbrain were isolated and dissected into left and right sides and used for RNA-seq sequencing.

For the fish collected in the predatory state (Lee et al. 2017), behavioral laterality (foraging preference = handedness) of the 12 juvenile *P. microlepis* was first determined. Briefly, for each fish, foraging behavior was monitored by counting the number of attacks to the left and/or right flanks on a single goldfish until a total of 20 attacks per individual were reached. The foraging laterality index (FLI) [FLI_foraging_ = (number of attacks_right_ − number of attacks_left_)/(number of attacks_right_ + number of attacks_left_)] was calculated individually (Facchin et al. 1999). A negative value of FLI indicates an R-morph fish, whereas a positive value indicates a L-morph fish. Brians of these experimental fish were isolated immediately after the end of the foraging trials. Seven different brain regions (telencephalon-L and -R, optic tectum-L and -R, hypothalamus-L and -R, and cerebellum) were dissected for each fish and used for RNA-seq sequencing.

### Read Filtering and Mapping

Adapters were removed from raw reads using Trimmomatic v0.39 (Bolger et al. 2014). The processed reads were then aligned to the *P. microlepis* reference genome (Tian et al. 2025) using STAR v2.7.3a (Dobin et al. 2013). Gene-level expression was quantified with RSEM v1.3.1 (Li and Dewey 2011), which calculated the number of reads mapped to each *P. microlepis* gene for each sample as the sum of the posterior probability that each read originated from the transcript (ie “expected counts”).

### Analysis of Differential Expression in Both Resting and Predatory States

To visualize the overall variation in expression patterns of the two datasets, we applied multidimensional scaling (MDS) using rlog-transformed, normalized gene counts as input. These counts were generated with DESeq2 (Love et al. 2014) in R v4.1.3 and analyzed with the *cmdscale* function from the R package *stats* (R Core Team 2021). Differentially expressed genes were identified through Wald tests, conducted separately for each brain region to compare brain regions and dominant versus nondominant conditions. Genes with a Benjamini-Hochberg corrected *P*-value < 0.05, as implemented in DESeq2, were considered significantly differentially expressed. To avoid potential batch effects, including those arising from differences in sequencing platform and sequencing depth between the two studies, differential expression and subsequent alternative splicing analyses were performed independently for each dataset, with comparisons restricted to the set of candidate genes.

### Analysis of Alternative Splicing in Both Resting and Predatory States

Alternative splicing events detection, quantification, and differential splicing analysis were conducted using rMATS-turbo (v4.1.0) (Wang et al. 2024). Five types of splicing patterns were detected: skipped exon (SE), alternative 5′ splice sites (A5SS), alternative 3′ splice sites (A3SS), mutually exclusive exons (MXE), and retained intron (RI). BAM files generated by STAR were used as input for rMATS-turbo. Supporting read counts were counted using both junction and exon reads. Exon inclusion levels were calculated as Percent Spliced In (PSI) values, ranging from 0 to 1, representing the ratio of reads supporting the inclusion isoform to the total reads. Differentially spliced events were defined using a false discovery rate (FDR) threshold <0.05 and PSI level >0.05. Visualization of alternative splicing events of interest was performed using rmats3sashimiplot software.

### Prediction of Potential Functional Differences Between Different Isoforms

To evaluate whether alternative splicing introduced premature stop codons (PTCs) and thereby potentially triggered nonsense-mediated decay (NMD), we examined the genome annotation of *P. microlepis* (Tian et al. 2025). Specifically, we assessed whether the length of the alternatively spliced exon was a multiple of three, whether splicing altered exon boundaries, and whether such changes introduced a new stop codon. When a new PTC was identified, we further determined whether it was located more than approximately 50 to 55 nt upstream of the final exon–exon junction, a commonly used criterion for predicting NMD activation (Nagy and Maquat 1998).

For protein domain prediction, we used the SMART tool (Letunic and Bork 2026) with the Pfam database (Mistry et al. 2021) selected. Protein structures of different splice isoforms were predicted using AlphaFold3 (Abramson et al. 2024) and visualized in ChimeraX (Meng et al. 2023).

### Functional Enrichment Analysis

We analyzed the potential functions of alternative spliced genes by extracting protein sequences from all identified genes in the *P. microlepis* reference genome (Tian et al. 2025) and identifying overrepresented Gene Ontology (GO) terms through hypergeometric tests using the KOBAS v.3.0 server (Bu et al. 2021). To account for multiple testing, we applied the Benjamini-Hochberg FDR correction, considering GO terms with an FDR-adjusted *P*-value < 0.01 as significantly enriched. Furthermore, we queried a curated list of fish genes (Herrera et al. 2025) to identify genes of interest and examine their functions and associated pathways, with particular attention to their roles in fish.

### Statistical Analysis of Sharing Among Gene Sets

We calculated the probability that alternative spliced genes between hemispheres were significantly shared among the three brain regions more often or less often than expected by chance with exact multiset intersection tests implemented in the SuperExactTest package (Wang et al. 2015). To determine if there were more or fewer genes that were significantly shared between two brain regions than would be expected by chance, we performed hypergeometric tests using scipy.stats function in Python (Virtanen et al. 2020).

## Results

### Global Expression Pattern of Alternative Splicing

To assess the relative abundance of alternative splicing and compare the expression patterns of differential expression and alternative splicing across different brain regions and behavioral states, we mapped brain transcriptome data from resting (Takeuchi et al. 2018) and predatory (Lee et al. 2017) states to the newly released chromosome-level reference genome (Tian et al. 2025). The first dataset included 88 transcriptomes from the left and right hemispheres of three brain regions in both L-eft and R-right morph individuals (morphs defined by external head morphology); all were obtained from fish that had been kept calm and isolated from other fish beforehand (hereafter referred to as “the resting state”). The second dataset (hereafter “the predatory state”) included 81 transcriptomes from the left and right hemispheres of four brain regions—three of which have anatomically discernible hemispheres—dissected immediately after a feeding attack in both L- and R-morph fish (with quantitative laterality measurement through behavioral experiments). Five brain regions are included in this study: the telencephalon, optic tectum, cerebellum, hypothalamus, and hindbrain. The telencephalon and optic tectum were covered in both studies (Fig. 1). The telencephalon is considered the primary center for cognition, decision making and other complex behaviors (Davis et al. 1981), is one of the main foci in this study, while the optic tectum is the dominant visual processing center in fish; it governs vision and prey capture (Northcutt 1983). The cerebellum coordinates movements and balance (Rodriguez et al. 2005), the hypothalamus regulates basic survival functions (Peter and Fryer 1983), and the hindbrain is responsible for autonomic and reflex control (Bass and Baker 1997). The results from both datasets indicated that at both the differential expression and alternative splicing levels, the brain regions were clearly separated and exhibited consistent patterns, with hemispheres of the same brain region clustering together and the two physiological states of the same brain region separating to some extent (Fig. 1).

**Fig. 1.**
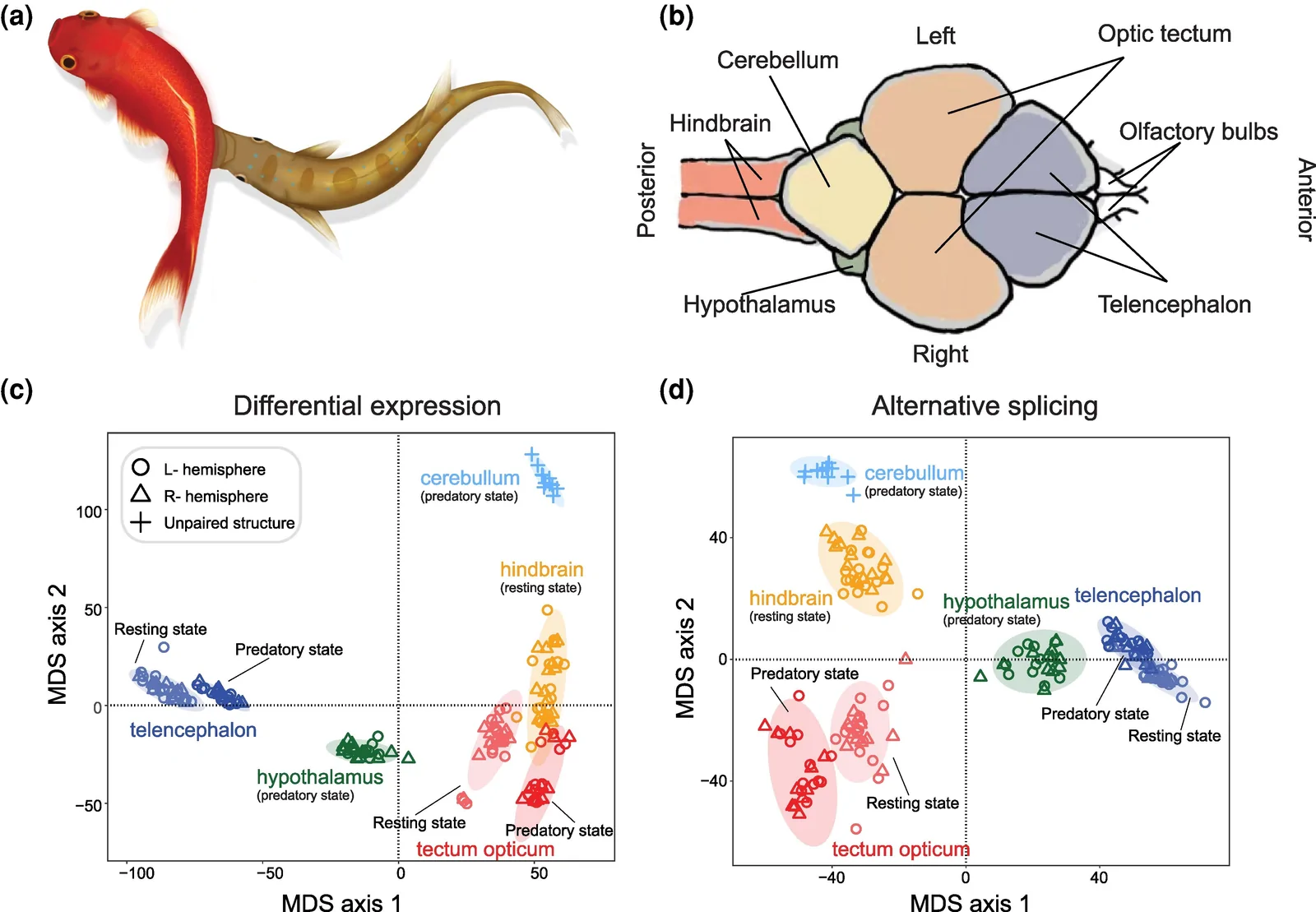
Illustration of lateralized foraging behavior in scale-eating cichlid fish and the brain regions analyzed in this study. a) Schematic of an L-morph individual attacking the right side of its prey. The illustration was created by Weiwei Li, based on an actual predatory event reported in Tian et al. 2024. b) Dorsal view of the brain regions examined. c) Multi-dimensional scaling (MDS) plot of gene expression from brain regions collected under resting and predatory states. d) MDS plot of gene splicing from brain regions collected under resting and predatory states. Tissues from left hemispheres are represented as circles, tissues from right hemispheres as triangles, and tissues from unpaired brain structures as crosses.

A large number of genes exhibited alternative splicing in pairwise comparisons of brain regions (with left and right hemispheres pooled) after multiple testing corrections (Tables 1 and 2). Notably, the number of alternatively spliced genes identified in the resting state and the predatory state differed significantly. In the resting state, the largest number of alternatively spliced genes was observed between the telencephalon and optic tectum (491 genes), followed by the hindbrain-telencephalon comparison (325 genes) and the hindbrain-optic tectum comparison (258 genes) (Table 1). In the predatory state, the highest number of alternatively spliced genes was also identified between the telencephalon and optic tectum (1,280 genes), while the lowest number was found between the hypothalamus and the telencephalon (Table 2). We further investigated the proportions of different alternative splicing patterns—alternative 3′ splice sites, alternative 5′ splice sites, mutually exclusive exons, retained intron and skipped exon—across all brain regions under both states, and found that the exon skipping is the predominant type (Fig. S1), consistent with its highest frequency in other vertebrates (Kim et al. 2007).

**Table 1:** Number of alternatively spliced genes between brain regions in the resting state, irrespective of individual lateralized behavior or brain hemisphere

Brain regions	Telencephalon	Hindbrain
Optic tectum	491	258
Telencephalon	…	325

**Table 2:** Number of alternatively spliced genes between brain regions in the predatory state, irrespective of individual lateralized behavior or brain hemisphere

Brain regions	Telencephalon	Hypothalamus	Cerebellum
Optic tectum	1,280	1,087	1,153
Telencephalon	…	606	1,190
Hypothalamus	…	…	1,199

### Links Between Behavioral Laterality and Alternative Splicing in the Brain in the Resting State

We first examined the genes exhibiting alternative splicing in the brain in the resting state by comparing the alternative splicing differences between dominant and nondominant hemispheres (ie genes spliced differently in the left hemisphere compared to the right hemisphere in the L-morph, and *vice versa*), across the three brain regions studied. The number of alternatively spliced genes varied across different brain regions, with the highest number of significant alternatively spliced genes (61 genes, 1.94% of all spliced genes) in the telencephalon, followed by the optic tectum and hindbrain (47 and 18 genes, 1.26% and 0.51% of all spliced genes, respectively). The number of alternatively spliced genes shared among the three brain regions was notably low (Exact Multi-set Intersection [EMI] *P*-value > 0.05; Fig. S2), suggesting functional specificity across different brain regions. We further assessed the genes with the most significant splicing differences (based on exon inclusion level differences) between dominant and nondominant hemispheres in each of the three brain regions (Table S1). The genes we have identified serve a variety of functions, including being involved in membrane transport and secretion (eg *SLC35G1*, *GPAA1*), as well as functions related to epigenetic regulation and transcription (eg *KDM4C*). Several genes associated with neurodevelopment were also identified. *ADGRL2* (adhesion G protein-coupled receptor L2) exhibited significant splicing differences in the telencephalon, with a 19% difference in exon inclusion levels between the dominant and nondominant hemispheres (Table S1). *RBFOX1* (RNA binding fox-1 homolog 1) also exhibited notable splicing variation in the optic tectum, displaying a 22% higher exon inclusion on the dominant side (Table S1).

### Links Between Behavioral Laterality and Alternative Splicing in the Brain Under the Predatory State

Next, we examined the links between alternative splicing events and behavioral laterality under the predatory state. We compared the alternative splicing differences between the dominant and nondominant hemispheres across three brain regions, identifying 119 genes (2.30% of all spliced genes) in the hypothalamus, 106 (1.89% of all spliced genes) in the optic tectum, and 89 (1.82% of all spliced genes) in the telencephalon. Most of these genes were tissue-specific, with only a small proportion (18 genes) shared across multiple brain regions (Exact Multi-set Intersection [EMI] *P*-value > 0.05; Fig. S3). In addition, we further explored the association of hemispheric splicing differences with the level (not just the direction) of behavioral laterality, which was quantified from a series of behavioral experiments. We found a comparable proportion of alternatively spliced genes significantly associated with lateralized behavior across brain regions: 38 of 119 alternatively spliced genes (31.93%) in the hypothalamus, 51 of 106 alternatively spliced genes (48.11%) in the optic tectum and 41 of 89 alternatively spliced genes (46.07%) in the telencephalon. Among these, microtubule-related genes *TTLL5* (tubulin tyrosine ligase-like 5) and *MARK3* (microtubule affinity-regulating kinase 3) were differentially spliced in the optic tectum and hypothalamus, respectively, and were strongly associated with behavioral laterality (*r* = 0.92 and 0.73; Tables S2 and S3). Additionally, two kinesin-related genes, *KIF1A* (kinesin family member 1A), shown in Fig. 2a, and *KIFC3* (kinesin family member C3) in the telencephalon were highly correlated with behavioral laterality (*r* = −0.76 and *r* = −0.91, respectively) (Table S4). Several neurodevelopment-related genes, including *RIMS2* (regulating synaptic membrane exocytosis 2, Fig. 2b) and *BPTF* (bromodomain PHD finger transcription factor, Fig. 2c), as well as synapse-related genes such as *GRIN1*, *NLGN1*, and *BDNF*, were identified (Tables S2 to S4). Regarding the two brain regions examined in both physiological states (resting and predatory), no genes were shared between the different states of the optic tectum, and only one gene (*RIMS2*) was common to the telencephalon in both behavioral states, but through different splicing events (Fig. S4), suggesting great differences of gene splicing in different states.

**Fig. 2.**
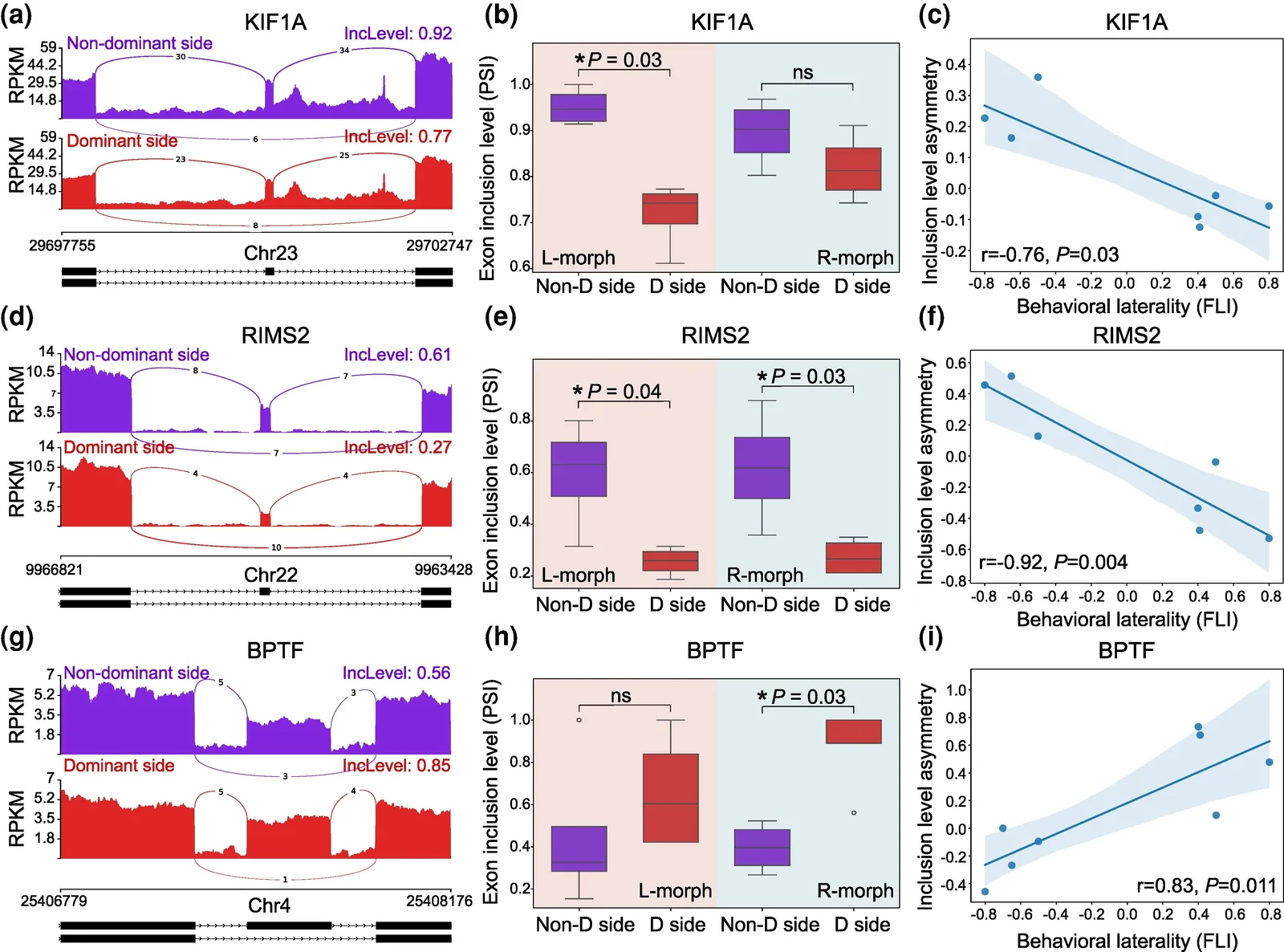
Representative candidate genes significantly associated with the degree of lateralized behavior. a) Difference in alternative splicing, measured by exon inclusion level, between the nondominant and dominant sides for the *KIF1A* gene. b) Comparison of alternative splicing differences between nondominant and dominant sides in L-morph and R-morph individuals. Non-D and D sides denote the nondominant and dominant sides, respectively. c) Correlation between behavioral laterality and exon inclusion level for *KIF1A*; Pearson correlation coefficient (*r*) and corresponding *P*-value are indicated. d–f) Same analyses as in (a–c), shown for *RIMS2*. g–i) Same analyses as in (a–c), shown for *BPTF*. ns: not significant; *: *P* < 0.05.

For these high-priority candidate genes, including *KIF1A*, *RIMS2*, and *BPTF*, our analysis showed that the alternatively spliced regions are located within coding regions and are translated into proteins without introducing nonsense-mediated decay. For *KIF1A*, the alternatively spliced exon is located within DUF3694, a Pfam-predicted domain (see Methods) found in some kinesin-like proteins and in Rab GTPase-activating protein 1, suggesting potential functional conservation. Protein structure prediction further suggested that inclusion of this exon alters the conformation of the DUF3694 domain by bringing two β-sheet strands into closer proximity (Fig. S5). For *RIMS2* and *BPTF*, the alternatively spliced regions are neither located within nor generate known domains, making functional predictions difficult. Protein structure prediction indicated that the alternatively spliced exons are located in disordered loops, suggesting that they may potentially alter the relative positioning of domains without directly contributing to domain formation (Figs. S6 and S7).

### Functional Enrichment of Alternatively Spliced genes

To investigate the potential functions of these alternatively spliced genes, we performed functional enrichment analyses on the identified gene sets. Among the alternatively spliced genes differing in the dominant and nondominant hemispheres of fish in the resting state, most of the enriched pathways were brain region-specific. Only one pathway (cytoplasm, GO:0005737) was shared across all three brain regions, while seven pathways were shared between two regions (Fig. 3a). Most of these pathways are related to cell structure (such as nucleus, cytosol, histone methyltransferase complex) and basic cellular functions (protein binding and RNA binding). No pathways were identified that could be directly linked to behavior or neurodevelopment. We further examined the brain region-specific pathways. Notably, compared to the optic tectum and hindbrain, the telencephalon encompasses numerous pathways related to neuronal development and synaptic regulation (Fig. S8).

**Fig. 3.**
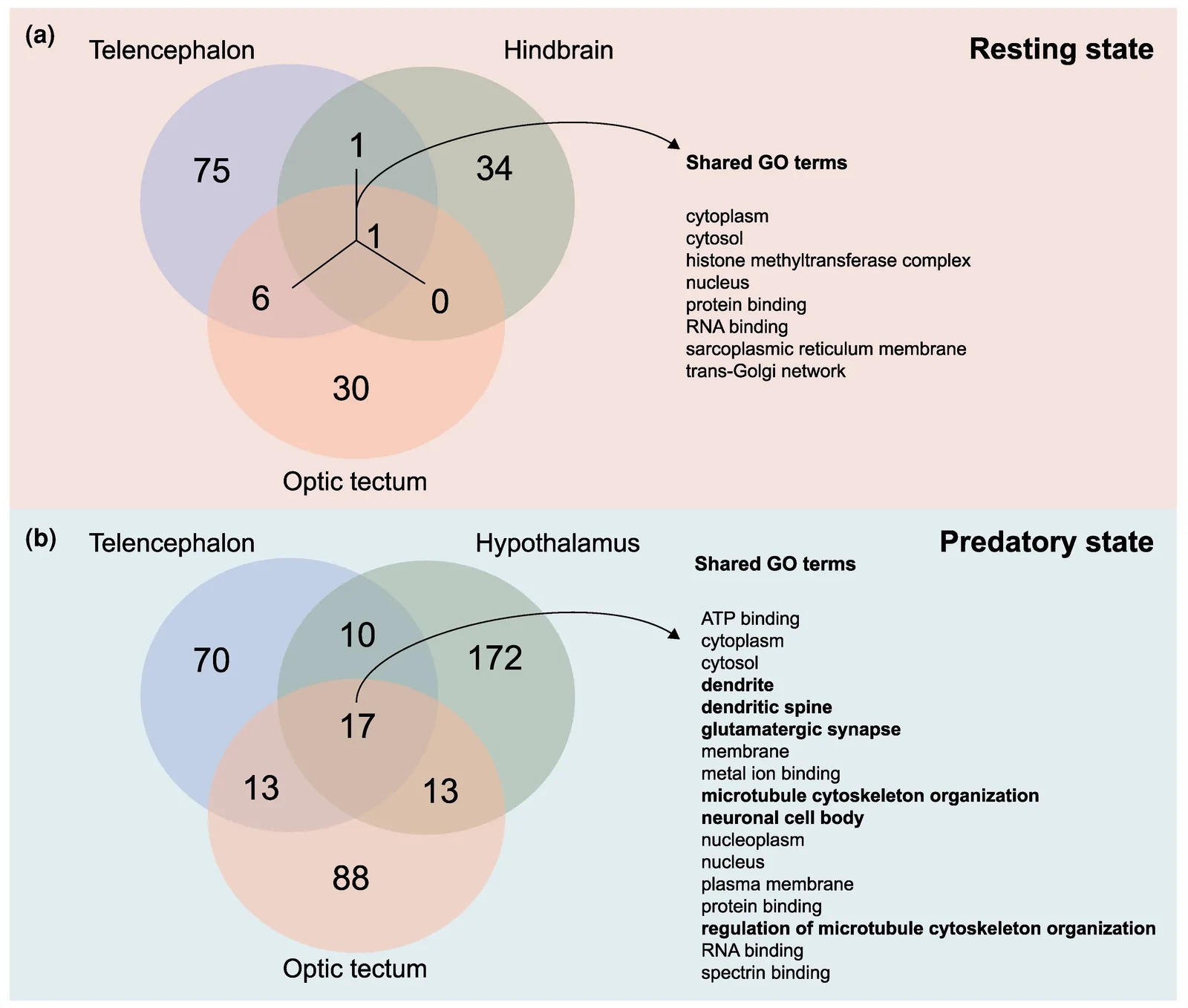
Functional enrichment of genes exhibiting alternatively splicing. a) Venn diagram illustrating the overlap of Gene Ontology (GO) terms associated with alternatively spliced genes across three brain regions under resting state conditions. b) Venn diagram illustrating the overlap of GO terms associated with alternatively spliced genes across the same brain regions under predatory state conditions. GO terms related to nervous system or microtubule are highlighted in bold. All pathways shown in the figure are statistically significant, and the pathways shown in bold indicate those related to the biological functions of interest.

Similarly, we analyzed the functional enrichment of alternatively spliced genes in the hemispheres of fish following predation activity. Regarding the alternatively spliced genes correlated with behavioral laterality, 17 pathways were shared across all three brain regions (Fig. 3b), statistically linking the function to handedness. These shared pathways are involved in neuronal structure and function (GO:000425, GO:004197, and GO:004025), synaptic transmission and plasticity (GO:0098978), cytoskeleton and microtubule regulation (GO:0000226 and GO:0070507), molecular binding and interaction (GO:0005524, GO:0046872, GO:0005515, and GO:00072), and cellular structure and localization (GO:000577, GO:0005829, GO:0005654 and GO:000564). Among them, pathways such as microtubule cytoskeleton organization (GO:0000226) and dendrite spine (GO:0043197) were also identified in a recent large-scale association study of human brain asymmetry (only four pathways reached significance) (Sha et al. 2021b) and in a single-cell transcriptomic analysis of the mouse left-right organizer, the embryonic structure where left-right patterning is set (Bellchambers et al. 2023), respectively. Regarding previously described handedness-related pathways (Cuellar-Partida et al. 2021; Bellchambers et al. 2023; Wang et al. 2023) enriched in each tissue, the most relevant pathways were clustered in the telencephalon and optic tectum (Fig. S9). A total of 29 pathways are shared in two tissues and are linked to various functions that may influence handedness, including axonal transport and guidance (GO:0008089 and GO:0007411), cytoskeleton dynamics and movement (GO:0007018, GO:0005874, and GO:0070507), and synaptic structure and function (GO:0048788, GO:0043083, and GO:0097091) (Fig. S10).

### Comparison Between Differential Expression and Alternative Splicing

Next, we compared the numbers of *differential expression* and *alternative splicing* across brain regions in the two states. Using our newly assembled genome and updated annotations, we reanalyzed the differential expression in these samples. In the resting state, consistent with a previous study (Takeuchi et al. 2018), we did not detect any genes associated with left-right lateralization. In the predatory state, a simple comparison between the dominant and nondominant sides of the brain likewise did not identify any genes that remained statistically significant after multiple-testing correction, also in agreement with previous work (Lee et al. 2017). However, when we considered lateralized behavior as a quantitative trait rather than a binary trait (lefty or righty), and correlated gene expression with behavioral laterality, the hypothalamus showed the largest number of differentially expressed genes (514), followed by the telencephalon and optic tectum (222 and 182, respectively) (Tables S5 to S7). We then asked whether differential expression and alternative splicing act on the same genes and found only limited overlap: one gene each in the telencephalon, optic tectum, and hypothalamus exhibited both differential expression and alternative splicing. These results appear to suggest that differential expression and alternative splicing largely involve distinct sets of genes.

We next investigated whether the same brain region shared common GO pathways across different states. Interestingly, in clear contrast to the optic tectum, the telencephalon showed 31 pathways shared between the two states, approximately half of which were related to the nervous system or microtubule-associated pathways (Fig. 4). In contrast, only seven pathways were shared between the two states in the optic tectum, and all were enriched for basic biological functions (Fig. 4).

**Fig. 4.**
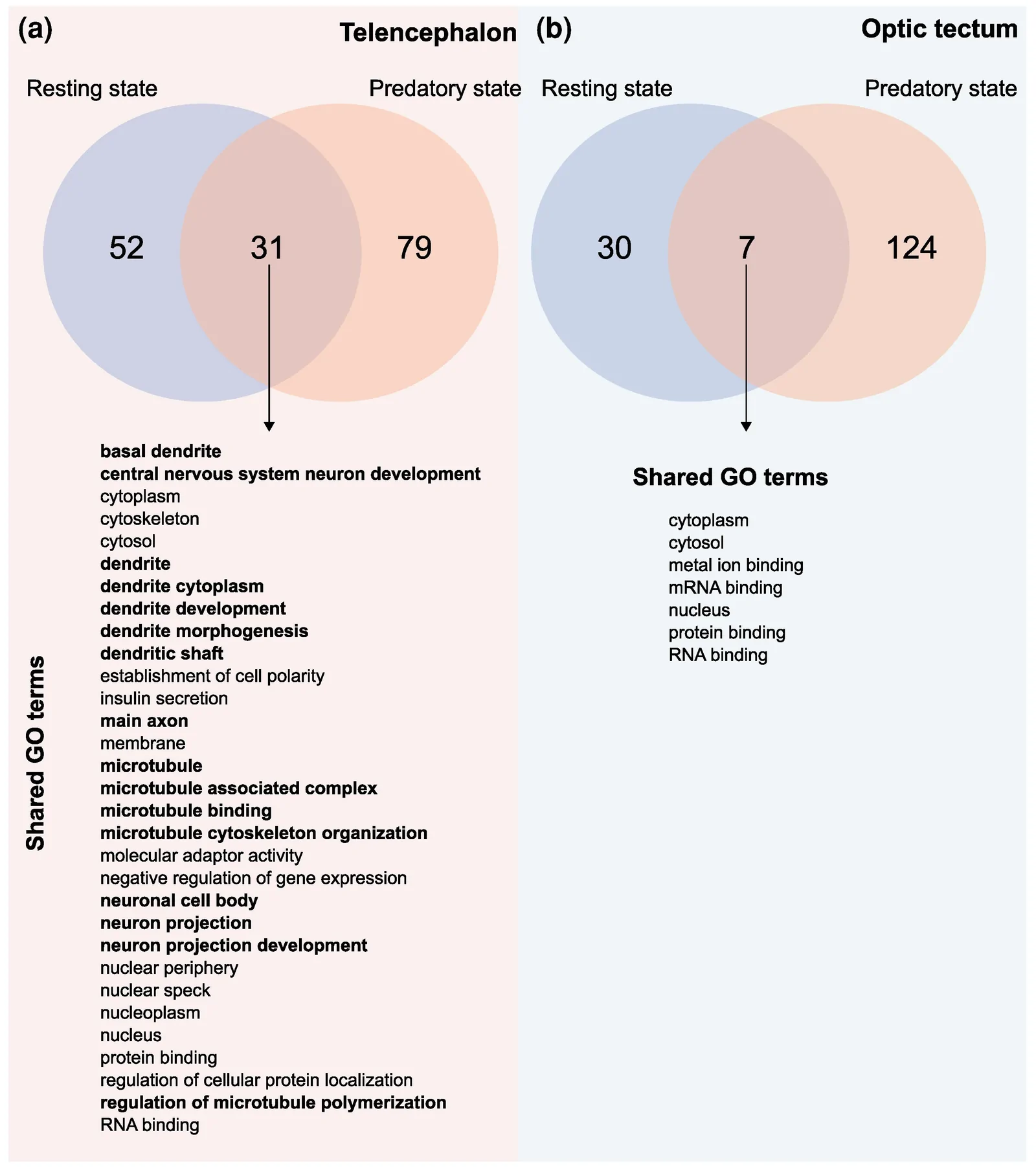
Comparison of functional enrichment for genes undergoing alternative splicing under two physiological states. In the telencephalon (a) and optic tectum (b), GO terms shared between the resting state and the predatory state. All pathways shown in the figure are statistically significant, and the pathways shown in bold indicate those related to the biological functions of interest.

## Discussion

In this study, we investigated the roles of differential expression and alternative splicing in behavioral laterality (handedness) in the bimodally asymmetric scale-eating cichlid *Perissodus microlepis*, a textbook example in evolutionary biology for negative frequency-dependent selection. Our results revealed that both alternative splicing and differential expression showed consistent, brain region-specific patterns. Limited overlap between resting and predatory states suggests state-specific splicing activity. During predation, functional profiles were more consistent across brain regions, with enrichment in nervous and microtubule-related pathways, especially in the telencephalon, the cognitive center of the vertebrate brain, and in the optic tectum, the visual-motor integration hub of the midbrain. In contrast, neurodevelopmental and synaptic pathways were enriched only in the telencephalon during rest. Notably, we identified several genes whose proportion of alternatively spliced transcripts was strongly correlated with behavioral laterality, linking the splicing regulation to handedness.

Our results revealed that only the telencephalon exhibited increased enrichment of pathways associated with handedness and the nervous system, both in resting and predatory states (Fig. 4). This highlights the telencephalon's pivotal role in lateralized behavior, also in this species of fish, which exhibits extremely lateralized predation behavior and asymmetrical head morphology. As the cognitive center of the vertebrate brain (Salas et al. 2003), the telencephalon is widely recognized for its involvement in advanced perceptual and cognitive functions, including learning, initiation of voluntary movements, and social-reproductive behaviors (Butler and Hodos 2005; Overmier and Patten 2013; Ganz et al. 2014). Lesion studies in teleost fish further support its involvement in spatial learning and memory (Portavella et al. 2002; Salas et al. 2006). Based on these findings, we hypothesized that the telencephalon plays a more prominent role in lateralized predatory behavior compared to other brain regions, potentially through its cognitive roles and activity during both predatory and resting states. Additionally, the optic tectum demonstrated significant enrichment of nervous system-related pathways during predation. The optic tectum, a central hub for visuomotor transformation across vertebrates (Kaas 1997; Cohen and Castro-Alamancos 2010), is primarily responsible for generating real-time spatial representations based on visual input (Northmore 2011). This aligns with our observation that enrichment of behavioral and nervous pathways was evident only in the predatory but not the resting state, showing clear hemispheric asymmetry in alternative splicing.

Our study found that, compared to the resting state, the predatory state exhibited a greater number of alternatively spliced genes and enriched pathways related to handedness and nervous systems—a possible indication that this process might play a role in “handed” behavior. In the resting state, most of the enriched pathways were associated with fundamental cellular functions, such as those involving the cytosol, nucleus, and protein binding. In contrast, genes showing interhemispheric alternative splicing during the predatory state were enriched for pathways related to neuronal structure and synaptic function, including dendrites, dendritic spines, and glutamatergic synapses. For instance, the proportion of alternatively spliced transcripts of genes such as *GRIN1*, *NLGN1*, and *BDNF,* which are known to play important roles in synapse formation and maintenance (Bramham and Messaoudi 2005; Wittenmayer et al. 2009; Zehavi et al. 2017), was significantly correlated with the degree of behavioral lateralization across individuals. Additionally, we observed enrichment in two microtubule-related pathways: microtubule cytoskeleton organization and regulation of microtubule cytoskeleton organization. A recent review has highlighted the role of microtubules in human handedness (Ocklenburg et al. 2025), and the same pathway—microtubule cytoskeleton organization—was identified in a recent large-scale association study (GWAS) of human brain asymmetry, in which only four pathways reached significance (Sha et al. 2021b). Although we did not identify any of the specific genes previously reported in large-scale human GWAS studies (Cuellar-Partida et al. 2021; Sha et al. 2021a; Sha et al. 2021b; Schijven et al. 2024), we did find several microtubule-associated genes with strong correlations to behavioral traits. For instance, expression of *TTLL5* (tubulin tyrosine ligase like 5) in the optic tectum showed a high correlation with lateralized behavior (*r* = 0.92), and although splicing differences for *MARK3* (microtubule affinity regulating kinase 3) in the hypothalamus were modest, its expression also correlated with lateralized behavior (*r* = 0.73). Furthermore, we identified several kinesin family genes (*KIFs*), which encode motor proteins responsible for transporting membranous organelles and macromolecules along microtubules—processes fundamental to cellular function (Hirokawa and Takemura 2004). Mutations or inactivation of *KIF* genes can disrupt intrinsic asymmetries; eg, approximately 50% of *KIF3A*- and *KIF3B*-deficient mice exhibit reversed heart looping (Nonaka et al. 1998; Marszalek et al. 1999; Takeda et al. 1999). In this study, alternative splicing of *KIF1A* and *KIFC3* was strongly associated with behavioral lateralization and showed significant splicing differences between dominant and nondominant hemispheres in both L- and R-morphs. Evidence from mice revealed that *KIF1A* is essential for hippocampal synaptogenesis and learning enhancement in an enriched environment (Kondo et al. 2012). *KIFC3* may play a role in the photoreceptor synapse in vertebrates (Hoang et al. 1999). Future research might provide deeper insights into these state-dependent differences by utilizing additional developmental time points and sensory deprivation experiments.

Here, we identified only a small number of genes showing both differential expression and alternative splicing, possibly suggesting that these processes are largely governed by distinct regulatory mechanisms and may affect partly different biological pathways. Consistent with this view, a cis-eQTL study in salmonid fish found that independent cis-regulatory regions affected either splicing or expression with <3.4% overlap (Jacobs and Elmer 2021). A similar conclusion is reached in a study on seasonal plasticity in butterflies, where gene expression and alternative splicing are found to play complementary roles in shaping plasticity (Steward et al. 2022). Comparative evidence across species further indicates that gene expression is generally conserved at the tissue level, whereas alternative splicing shows stronger species-specific patterning (Barbosa-Morais et al. 2012; Merkin et al. 2012; Singh and Ahi 2022), implying that splicing may diverge more rapidly over short evolutionary timescales and contribute disproportionately to species-specific traits such as the lateralized foraging behavior in our fish. Alternative splicing has been linked to diverse physiological processes, including the regulation of behavior. For example, alternative splicing of a single transcription factor can drive selfish reproductive behavior in honeybee workers (Jarosch et al. 2011), and tame versus aggressive rats show pronounced differences in gene splicing across brain regions (Babenko et al. 2017). In zebrafish, hunger-induced orexin enhances aggression through biased alternative splicing of the *AMPA* receptor gene in the interpeduncular nucleus (Nakajo et al. 2020). Given the growing utility of fish models in neuroscience (Legare et al. 2023), especially single-cell sequencing of entire brain regions (Pandey et al. 2018; Pandey et al. 2023; Ferrero et al. 2025), future application of single-cell and spatial transcriptomics in our system could help resolve left-right asymmetry in cellular composition, gene expression, and alternative splicing.

In summary, our findings suggest that alternative splicing, which plays a central role in post-transcriptional regulation, may be a key mechanism underlying brain functional asymmetries, such as handedness, and perhaps many other characteristics that are believed to be associated with hemispheric specialization, perhaps also or especially in mammals and primates like us. Our results highlight a set of candidate genes for further exploration in the context of lateralized behavior, brain asymmetry, and neurological disorders in humans.

## Supplementary Material

evag237_Supplementary_Data

## Data Availability

The RNA-seq datasets analyzed in this study were obtained from two previously published studies (Lee et al. 2017; Takeuchi et al. 2018) with the NCBI BioProject accession numbers PRJNA353066 and PRJDB6174. The reference genome used was NCBI assembly ASM5116744v1. All data processing programs were executed with default parameters unless otherwise specified in the *Materials and Methods* section. There were no custom scripts or code utilized in this study.

## References

[evag237-B1] Abramson J, et al Accurate structure prediction of biomolecular interactions with AlphaFold 3. Nature. 2024:630:493–500. 10.1038/s41586-024-07487-w. PMC11168924 38718835

[evag237-B2] Alhabsi A, Ling Y, Crespi M, Reddy ASN, Mahfouz M. Alternative splicing dynamics in plant adaptive responses to stress. Annu Rev Plant Biol. 2025:76:687–717. 10.1146/annurev-arplant-083123-090055. 39952682

[evag237-B3] Babenko VN, Bragin AO, Chadaeva IV, Markel AL, Orlov YL. Differential alternative splicing in brain regions of rats selected for aggressive behavior. Mol Biol. 2017:51:759–768. 10.1134/s002689331705003x. 29116075

[evag237-B4] Badano JL, Mitsuma N, Beales PL, Katsanis N. The ciliopathies: an emerging class of human genetic disorders. Annu Rev Genomics Hum Genet. 2006:7:125–148. 10.1146/annurev.genom.7.080505.115610. 16722803

[evag237-B5] Barbosa-Morais NL, et al The evolutionary landscape of alternative splicing in vertebrate species. Science. 2012:338:1587–1593. 10.1126/science.1230612. 23258890

[evag237-B6] Bass AH, Baker R. Phenotypic specification of hindbrain rhombomeres and the origins of rhythmic circuits in vertebrates. Brain Behav Evol. 1997:50:3–16. 10.1159/000113351. PMC3023276 9217990

[evag237-B7] Bellchambers HM, et al Single cell RNA analysis of the left-right organizer transcriptome reveals potential novel heterotaxy genes. Sci Rep. 2023:13:10688. 10.1038/s41598-023-36862-2. PMC10314903 37393374

[evag237-B8] Berlinghieri F, Panizzon P, Penry-Williams IL, Brown C. Laterality and fish welfare - a review. Appl Anim Behav Sci. 2021:236:105239. 10.1016/j.applanim.2021.105239.

[evag237-B9] Bolger AM, Lohse M, Usadel BJB. Trimmomatic: a flexible trimmer for Illumina sequence data. Bioinformatics. 2014:30:2114–2120. 10.1093/bioinformatics/btu170. PMC4103590 24695404

[evag237-B10] Bramham CR, Messaoudi E. BDNF function in adult synaptic plasticity: the synaptic consolidation hypothesis. Prog Neurobiol. 2005:76:99–125. 10.1016/j.pneurobio.2005.06.003. 16099088

[evag237-B11] Bu D, et al KOBAS-i: intelligent prioritization and exploratory visualization of biological functions for gene enrichment analysis. Nucleic Acids Res. 2021:49:W317–W325. 10.1093/nar/gkab447. PMC8265193 34086934

[evag237-B12] Butler AB, Hodos W. Comparative vertebrate neuroanatomy: evolution and adaptation. John Wiley & Sons; 2005.

[evag237-B13] Caspar KR, Pallasdies F, Mader L, Sartorelli H, Begall S. The evolution and biological correlates of hand preferences in anthropoid primates. Elife. 2022:11:e77875. 10.7554/eLife.77875. PMC9714969 36454207

[evag237-B14] Cohen JD, Castro-Alamancos MA. Neural correlates of active avoidance behavior in superior colliculus. J Neurosci. 2010:30:8502–8511. 10.1523/JNEUROSCI.1497-10.2010. PMC2905738 20573897

[evag237-B15] Coren S, Porac C. Fifty centuries of right-handedness: the historical record. Science. 1977:198:631–632. 10.1126/science.335510. 335510

[evag237-B16] Cuellar-Partida G, et al Genome-wide association study identifies 48 common genetic variants associated with handedness. Nat Hum Behav. 2021:5:59–70. 10.1038/s41562-020-00956-y. PMC7116623 32989287

[evag237-B17] Danaisawadi P, Asami T, Ota H, Sutcharit C, Panha S. A snail-eating snake recognizes prey handedness. Sci Rep. 2016:6:23832. 10.1038/srep23832. PMC4820687 27046345

[evag237-B18] Davis RE, Chase R, Morris J, Kaufman B. Telencephalon of the teleost Macropodus: experimental localization of secondary olfactory areas and of components of the lateral forebrain bundle. Behav Neural Biol. 1981:33:257–279. 10.1016/s0163-1047(81)92032-x.

[evag237-B19] Dobin A, et al STAR: ultrafast universal RNA-seq aligner. Bioinformatics. 2013:29:15–21. 10.1093/bioinformatics/bts635. PMC3530905 23104886

[evag237-B20] Facchin L, Bisazza A, Vallortigara G. What causes lateralization of detour behavior in fish? Evidence for asymmetries in eye use. Behav Brain Res. 1999:103:229–234. 10.1016/s0166-4328(99)00043-1. 10513591

[evag237-B21] Ferrero G, et al A single-cell transcriptomic atlas reveals resident dendritic-like cells in the zebrafish brain parenchyma. Elife. 2025:13:RP91427. 10.7554/eLife.91427.3. PMC12698085 41379882

[evag237-B22] Fliegauf M, Benzing T, Omran H. When cilia go bad: cilia defects and ciliopathies. Nat Rev Mol Cell Biol. 2007:8:880–893. 10.1038/nrm2278. 17955020

[evag237-B23] Frost GT . Tool behavior and the origins of laterality. J Hum Evol. 1980:9:447–459. 10.1016/0047-2484(80)90002-0.

[evag237-B24] Futuyma D . Evolution. Sinauer Associates; 2009.

[evag237-B25] Ganz J, et al Subdivisions of the adult zebrafish pallium based on molecular marker analysis. F1000Res. 2014:3:308. 10.12688/f1000research.5595.2. PMC4335597 25713698

[evag237-B26] Ghirlanda S, Vallortigara G. The evolution of brain lateralization: a game-theoretical analysis of population structure. Proc Biol Sci. 2004:271:853–857. 10.1098/rspb.2003.2669. PMC1691668 15255105

[evag237-B27] Goeke S, et al Alternative splicing of lola generates 19 transcription factors controlling axon guidance in Drosophila. Nat Neurosci. 2003:6:917–924. 10.1038/nn1105. 12897787

[evag237-B28] Gothié E, Richard DE, Berra E, Pages G, Pouysségur J. Identification of alternative spliced variants of human hypoxia-inducible factor-1α. J Biol Chem. 2000:275:6922–6927. 10.1074/jbc.275.10.6922. 10702253

[evag237-B29] Harris LJ . Footedness in parrots: three centuries of research, theory, and mere surmise. Can J Psychol. 1989:43:369–396. 10.1037/h0084228. 2519739

[evag237-B30] Hata H, Yasugi M, Takeuchi Y, Takahashi S, Hori M. Measuring and evaluating morphological asymmetry in fish: distinct lateral dimorphism in the jaws of scale-eating cichlids. Ecol Evol. 2013:3:4641–4647. 10.1002/ece3.849. PMC3867900 24363893

[evag237-B31] Hattori D, et al Robust discrimination between self and non-self neurites requires thousands of Dscam1 isoforms. Nature. 2009:461:644–648. 10.1038/nature08431. PMC2836808 19794492

[evag237-B32] Herrera M, et al From genes to pathways: a curated gene approach to accurate pathway reconstruction in teleost fish transcriptomics. J Exp Zool B Mol Dev Evol. 2025:344:303–317. 10.1002/jez.b.23299. 40296566

[evag237-B33] Herve PY, Crivello F, Perchey G, Mazoyer B, Tzourio-Mazoyer N. Handedness and cerebral anatomical asymmetries in young adult males. Neuroimage. 2006:29:1066–1079. 10.1016/j.neuroimage.2005.08.031. 16198126

[evag237-B34] Hirokawa N, Takemura R. Kinesin superfamily proteins and their various functions and dynamics. Exp Cell Res. 2004:301:50–59. 10.1016/j.yexcr.2004.08.010. 15501445

[evag237-B35] Hoang E, Bost-Usinger L, Burnside B. Characterization of a novel C-kinesin (KIFC3) abundantly expressed in vertebrate retina and RPE. Exp Eye Res. 1999:69:57–68. 10.1006/exer.1999.0671. 10375449

[evag237-B36] Hopkins WD . Neuroanatomical asymmetries and handedness in chimpanzees (Pan troglodytes): a case for continuity in the evolution of hemispheric specialization. Ann N Y Acad Sci. 2013:1288:17–35. 10.1111/nyas.12109. PMC3676728 23647534

[evag237-B37] Hopkins WD . A review of performance asymmetries in hand skill in nonhuman primates with a special emphasis on chimpanzees. Prog Brain Res. 2018:238:57–89. 10.1016/bs.pbr.2018.06.012. 30097203

[evag237-B38] Hori M . Frequency-dependent natural selection in the handedness of scale-eating cichlid fish. Science. 1993:260:216–219. 10.1126/science.260.5105.216. 17807183

[evag237-B39] Hulthén K, Heinen-Kay JL, Schmidt DA, Langerhans RB, Smiseth P. Predation shapes behavioral lateralization: insights from an adaptive radiation of livebearing fish. Behav Ecol. 2021:32:1321–1329. 10.1093/beheco/arab098.

[evag237-B40] Hunter CE, Xing Y. The splice of life: how alternative splicing shapes regulatory and phenotypic evolution. EMBO J. 2026:45:1878–1888. 10.1038/s44318-025-00666-z. PMC12992808 41545761

[evag237-B41] Jacobs A, Elmer KR. Alternative splicing and gene expression play contrasting roles in the parallel phenotypic evolution of a salmonid fish. Mol Ecol. 2021:30:4955–4969. 10.1111/mec.15817. PMC8653899 33502030

[evag237-B42] Jarosch A, Stolle E, Crewe RM, Moritz RF. Alternative splicing of a single transcription factor drives selfish reproductive behavior in honeybee workers (*Apis mellifera*). Proc Natl Acad Sci U S A. 2011:108:15282–15287. 10.1073/pnas.1109343108. PMC3174643 21896748

[evag237-B43] Kaas JH . Topographic maps are fundamental to sensory processing. Brain Res Bull. 1997:44:107–112. 10.1016/s0361-9230(97)00094-4. 9292198

[evag237-B44] Kim E, Magen A, Ast G. Different levels of alternative splicing among eukaryotes. Nucleic Acids Res. 2007:35:125–131. 10.1093/nar/gkl924. PMC1802581 17158149

[evag237-B45] Kondo M, Takei Y, Hirokawa N. Motor protein KIF1A is essential for hippocampal synaptogenesis and learning enhancement in an enriched environment. Neuron. 2012:73:743–757. 10.1016/j.neuron.2011.12.020. 22365548

[evag237-B46] Kusche H, Lee HJ, Meyer A. Mouth asymmetry in the textbook example of scale-eating cichlid fish is not a discrete dimorphism after all. Proc Biol Sci. 2012:279:4715–4723. 10.1098/rspb.2012.2082. PMC3497103 23055070

[evag237-B47] Lee HJ, et al Genetic support for random mating between left and right-mouth morphs in the dimorphic scale-eating cichlid fish *Perissodus microlepis* from Lake Tanganyika. J Fish Biol. 2010:76:1940–1957. 10.1111/j.1095-8649.2010.02620.x. 20557648

[evag237-B48] Lee HJ, et al Lateralized feeding behavior is associated with asymmetrical neuroanatomy and lateralized gene expressions in the brain in scale-eating cichlid fish. Genome Biol Evol. 2017:9:3122–3136. 10.1093/gbe/evx218. PMC5737854 29069363

[evag237-B49] Lee HJ, Heim V, Meyer A. Genetic and environmental effects on the morphological asymmetry in the scale-eating cichlid fish, *Perissodus microlepis*. Ecol Evol. 2015:5:4277–4286. 10.1002/ece3.1691. PMC4667837 26664678

[evag237-B50] Lee HJ, Heim V, Meyer A. Genetic evidence for prevalence of alloparental care in a socially monogamous biparental cichlid fish, *Perissodus microlepis*, from Lake Tanganyika supports the “selfish shepherd effect” hypothesis. Ecol Evol. 2016:6:2843–2853. 10.1002/ece3.2089. PMC4863010 27217943

[evag237-B51] Lee HJ, Kusche H, Meyer A. Handed foraging behavior in scale-eating cichlid fish: its potential role in shaping morphological asymmetry. PLoS One. 2012:7:e44670. 10.1371/journal.pone.0044670. PMC3435272 22970282

[evag237-B52] Legare A, Lemieux M, Desrosiers P, De Koninck P. Zebrafish brain atlases: a collective effort for a tiny vertebrate brain. Neurophotonics. 2023:10:044409. 10.1117/1.NPh.10.4.044409. PMC10541682 37786400

[evag237-B53] Letunic I, Bork P. SMART v10: three decades of the protein domain annotation resource. Nucleic Acids Res. 2026:54:D499–D503. 10.1093/nar/gkaf1023. PMC12807790 41062452

[evag237-B54] Li B, Dewey CN. RSEM: accurate transcript quantification from RNA-Seq data with or without a reference genome. BMC Bioinformatics. 2011:12:323. 10.1186/1471-2105-12-323. PMC3163565 21816040

[evag237-B55] Liu Y, et al Importance of pre-mRNA splicing and its study tools in plants. Adv Biotechnol. 2024:2:4. 10.1007/s44307-024-00009-9. PMC11740881 39883322

[evag237-B56] Lonsdorf EV, Hopkins WD. Wild chimpanzees show population-level handedness for tool use. Proc Natl Acad Sci U S A. 2005:102:12634–12638. 10.1073/pnas.0505806102. PMC1194943 16105943

[evag237-B57] Love MI, Huber W, Anders S. Moderated estimation of fold change and dispersion for RNA-Seq data with DESeq2. Genome Biol. 2014:15:550. 10.1186/s13059-014-0550-8. PMC4302049 25516281

[evag237-B58] Marszalek JR, Ruiz-Lozano P, Roberts E, Chien KR, Goldstein LS. Situs inversus and embryonic ciliary morphogenesis defects in mouse mutants lacking the KIF3A subunit of kinesin-II. Proc Natl Acad Sci U S A. 1999:96:5043–5048. 10.1073/pnas.96.9.5043. PMC21813 10220415

[evag237-B59] Meng EC, et al UCSF ChimeraX: tools for structure building and analysis. Protein Sci. 2023:32:e4792. 10.1002/pro.4792. PMC10588335 37774136

[evag237-B60] Merkin J, Russell C, Chen P, Burge CB. Evolutionary dynamics of gene and isoform regulation in mammalian tissues. Science. 2012:338:1593–1599. 10.1126/science.1228186. PMC3568499 23258891

[evag237-B61] Michel GF, Babik I, Nelson EL, Campbell JM, Marcinowski EC. Evolution and development of handedness: an Evo-Devo approach. Prog Brain Res. 2018:238:347–374. 10.1016/bs.pbr.2018.06.007. 30097200

[evag237-B62] Mistry J, et al Pfam: the protein families database in 2021. Nucleic Acids Res. 2021:49:D412–D419. 10.1093/nar/gkaa913. PMC7779014 33125078

[evag237-B63] Nagy E, Maquat LE. A rule for termination-codon position within intron-containing genes: when nonsense affects RNA abundance. Trends Biochem Sci. 1998:23:198–199. 10.1016/s0968-0004(98)01208-0. 9644970

[evag237-B64] Nakajo H, et al Hunger potentiates the habenular winner pathway for social conflict by orexin-promoted biased alternative splicing of the AMPA receptor gene. Cell Rep. 2020:31:107790. 10.1016/j.celrep.2020.107790. 32579920

[evag237-B65] Nonaka S, et al Randomization of left-right asymmetry due to loss of nodal cilia generating leftward flow of extraembryonic fluid in mice lacking KIF3B motor protein. Cell. 1998:95:829–837. 10.1016/s0092-8674(00)81705-5. 9865700

[evag237-B66] Northcutt R . Evolution of the optic tectum in ray-finned fishes. In: Fish neurobiology. University of Michigan Press; 1983. p. 1–42.

[evag237-B67] Northmore D . The optic tectum. In: Encyclopedia of fish physiology: from genome to environment. Elsevier; 2011. p. 131–142.

[evag237-B68] Ocklenburg S, Gunturkun O. Handedness. Handb Clin Neurol. 2025:208:379–391. 10.1016/B978-0-443-15646-5.00016-6. 40074409

[evag237-B69] Ocklenburg S, Mundorf A, Peterburs J, Paracchini S. Genetics of human handedness: microtubules and beyond. Trends Genet. 2025:41:497–505. 10.1016/j.tig.2025.01.006. 39894687

[evag237-B70] Overmier JB, Patten RL. Teleost telencephalon and memory for delayed reinforcers. Psychobiology. 2013:10:74–78. 10.3758/bf03327010.

[evag237-B71] Pandey S, Moyer AJ, Thyme SB. A single-cell transcriptome atlas of the maturing zebrafish telencephalon. Genome Res. 2023:33:658–671. 10.1101/gr.277278.122. PMC10234298 37072188

[evag237-B72] Pandey S, Shekhar K, Regev A, Schier AF. Comprehensive identification and spatial mapping of habenular neuronal types using single-cell RNA-Seq. Curr Biol. 2018:28:1052–1065.e7. 10.1016/j.cub.2018.02.040. PMC6042852 29576475

[evag237-B73] Papadatou-Pastou M, et al Human handedness: a meta-analysis. Psychol Bull. 2020:146:481–524. 10.1037/bul0000229. 32237881

[evag237-B74] Peter R, Fryer J. Endocrine functions of the hypothalamus of actinopterygians. In: Fish neurobiology. University of Michigan Press; 1983. p. 165–201.

[evag237-B75] Portavella M, Vargas JP, Torres B, Salas C. The effects of telencephalic pallial lesions on spatial, temporal, and emotional learning in goldfish. Brain Res Bull. 2002:57:397–399. 10.1016/s0361-9230(01)00699-2. 11922997

[evag237-B76] Raffini F, Fruciano C, Meyer A. Gene(s) and individual feeding behavior: exploring eco-evolutionary dynamics underlying left-right asymmetry in the scale-eating cichlid fish *Perissodus microlepis*. Ecol Evol. 2018a:8:5495–5507. 10.1002/ece3.4070. PMC6010907 29938068

[evag237-B77] Raffini F, Fruciano C, Meyer A. Morphological and genetic correlates in the left–right asymmetric scale-eating cichlid fish of Lake Tanganyika. Biol J Linn Soc Lond. 2018b:124:67–84. 10.1093/biolinnean/bly024.

[evag237-B78] Raffini F, Meyer A. A comprehensive overview of the developmental basis and adaptive significance of a textbook polymorphism: head asymmetry in the cichlid fish *Perissodus microlepis*. Hydrobiologia. 2018:832:65–84. 10.1007/s10750-018-3800-z.

[evag237-B79] R Core Team . R: a language and environment for statistical computing. R Foundation for Statistical Computing; 2021.

[evag237-B80] Rodriguez F, et al Cognitive and emotional functions of the teleost fish cerebellum. Brain Res Bull. 2005:66:365–370. 10.1016/j.brainresbull.2004.11.026. 16144616

[evag237-B81] Rogers LJ . Brain lateralization and cognitive capacity. Animals (Basel). 2021:11:1996. 10.3390/ani11071996. PMC8300231 34359124

[evag237-B82] Rogers LJ, Andrew R. Comparative vertebrate lateralization. Cambridge University Press; 2002.

[evag237-B83] Rogers LJ, Zucca P, Vallortigara G. Advantages of having a lateralized brain. Proc Biol Sci. 2004:271:S420–S422. 10.1098/rsbl.2004.0200. PMC1810119 15801592

[evag237-B84] Salas C, et al Neuropsychology of learning and memory in teleost fish. Zebrafish. 2006:3:157–171. 10.1089/zeb.2006.3.157. 18248258

[evag237-B85] Salas C, Broglio C, Rodriguez F. Evolution of forebrain and spatial cognition in vertebrates: conservation across diversity. Brain Behav Evol. 2003:62:72–82. 10.1159/000072438. 12937346

[evag237-B86] Schaub A, Glasmacher E. Splicing in immune cells-mechanistic insights and emerging topics. Int Immunol. 2017:29:173–181. 10.1093/intimm/dxx026. PMC5890895 28498981

[evag237-B87] Schijven D, Soheili-Nezhad S, Fisher SE, Francks C. Exome-wide analysis implicates rare protein-altering variants in human handedness. Nat Commun. 2024:15:2632. 10.1038/s41467-024-46277-w. PMC10987538 38565598

[evag237-B88] Sha Z, et al Handedness and its genetic influences are associated with structural asymmetries of the cerebral cortex in 31,864 individuals. Proc Natl Acad Sci U S A. 2021a:118:e2113095118. 10.1073/pnas.2113095118. PMC8617418 34785596

[evag237-B89] Sha Z, et al The genetic architecture of structural left-right asymmetry of the human brain. Nat Hum Behav. 2021b:5:1226–1239. 10.1038/s41562-021-01069-w. PMC8455338 33723403

[evag237-B90] Singh P, Ahi EP. The importance of alternative splicing in adaptive evolution. Mol Ecol. 2022:31:1928–1938. 10.1111/mec.16377. 35094439

[evag237-B91] Steward RA, de Jong MA, Oostra V, Wheat CW. Alternative splicing in seasonal plasticity and the potential for adaptation to environmental change. Nat Commun. 2022:13:755. 10.1038/s41467-022-28306-8. PMC8825856 35136048

[evag237-B92] Stewart TA, Albertson RC. Evolution of a unique predatory feeding apparatus: functional anatomy, development and a genetic locus for jaw laterality in Lake Tanganyika scale-eating cichlids. BMC Biol. 2010:8:8. 10.1186/1741-7007-8-8. PMC2828976 20102595

[evag237-B93] Takeda S, et al Left-right asymmetry and kinesin superfamily protein KIF3A: new insights in determination of laterality and mesoderm induction by kif3A-/- mice analysis. J Cell Biol. 1999:145:825–836. 10.1083/jcb.145.4.825. PMC2133177 10330409

[evag237-B94] Takeuchi Y, Higuchi Y, Ikeya K, Tagami M, Oda Y. Experience-dependent learning of behavioral laterality in the scale-eating cichlid *Perissodus microlepis* occurs during the early developmental stage. Sci Rep. 2022:12:723. 10.1038/s41598-021-04588-8. PMC8760303 35031653

[evag237-B95] Takeuchi Y, Hori M, Oda Y. Lateralized kinematics of predation behavior in a Lake Tanganyika scale-eating cichlid fish. PLoS One. 2012:7:e29272. 10.1371/journal.pone.0029272. PMC3253053 22238598

[evag237-B96] Takeuchi Y, Ishikawa A, Oda Y, Kitano J. Lateralized expression of left-right axis formation genes is shared by adult brains of lefty and righty scale-eating cichlids. Comp Biochem Physiol Part D Genomics Proteomics. 2018:28:99–106. 10.1016/j.cbd.2018.07.002. 30007273

[evag237-B97] Tao Y, Zhang Q, Wang H, Yang X, Mu H. Alternative splicing and related RNA binding proteins in human health and disease. Signal Transduct Target Ther. 2024:9:26. 10.1038/s41392-024-01734-2. PMC10835012 38302461

[evag237-B98] Terrapon N, et al Molecular traces of alternative social organization in a termite genome. Nat Commun. 2014:5:3636. 10.1038/ncomms4636. 24845553

[evag237-B99] Tian X, Lee S, Tuckermann J, Meyer A. Bilateral asymmetry in craniofacial structures and kinematics of feeding attacks in the scale-eating cichlid fish, *Perissodus microlepis*. Zool Res. 2024:46:370–378. 10.24272/j.issn.2095-8137.2024.314. PMC12000126 40091531

[evag237-B100] Tian X, Li M, Meyer A. Insights into the genetic basis of bilateral head asymmetry in a scale-eating cichlid fish. Sci Adv. 2025:11:eadw4406. 10.1126/sciadv.adw4406. PMC12309687 40737413

[evag237-B101] Uhlen M, et al Proteomics. Tissue-based map of the human proteome. Science. 2015:347:1260419. 10.1126/science.1260419. 25613900

[evag237-B102] Ustaoglu P, et al Dynamically expressed single ELAV/Hu orthologue elavl2 of bees is required for learning and memory. Commun Biol. 2021:4:1234. 10.1038/s42003-021-02763-1. PMC8553928 34711922

[evag237-B103] Verta JP, Jacobs A. The role of alternative splicing in adaptation and evolution. Trends Ecol Evol. 2022:37:299–308. 10.1016/j.tree.2021.11.010. 34920907

[evag237-B104] Virtanen P, et al Scipy 1.0: fundamental algorithms for scientific computing in Python. Nat Methods. 2020:17:261–272. 10.1038/s41592-019-0686-2. PMC7056644 32015543

[evag237-B105] Wang J, Ma S, Yu P, He X. Evolution of human brain left-right asymmetry: old genes with new functions. Mol Biol Evol. 2023:40:msad181. 10.1093/molbev/msad181. PMC10473864 37561991

[evag237-B106] Wang M, Zhao Y, Zhang B. Efficient test and visualization of multi-set intersections. Sci Rep. 2015:5:16923. 10.1038/srep16923. PMC4658477 26603754

[evag237-B107] Wang Y, et al rMATS-turbo: an efficient and flexible computational tool for alternative splicing analysis of large-scale RNA-Seq data. Nat Protoc. 2024:19:1083–1104. 10.1038/s41596-023-00944-2. 38396040

[evag237-B108] Ward JP . Left hand advantage for prey capture in the Galago (Galago Moholi). Int J Comp Psychol. 2012:11:173–184. 10.46867/c41c78.

[evag237-B109] Wittenmayer N, et al Postsynaptic Neuroligin1 regulates presynaptic maturation. Proc Natl Acad Sci U S A. 2009:106:13564–13569. 10.1073/pnas.0905819106. PMC2726414 19628693

[evag237-B110] Wright CJ, Smith CWJ, Jiggins CD. Alternative splicing as a source of phenotypic diversity. Nat Rev Genet. 2022:23:697–710. 10.1038/s41576-022-00514-4. 35821097

[evag237-B111] Yeo G, Holste D, Kreiman G, Burge CB. Variation in alternative splicing across human tissues. Genome Biol. 2004:5:R74. 10.1186/gb-2004-5-10-r74. PMC545594 15461793

[evag237-B112] Zehavi Y, et al De novo GRIN1 mutations: an emerging cause of severe early infantile encephalopathy. Eur J Med Genet. 2017:60:317–320. 10.1016/j.ejmg.2017.04.001. 28389307

[evag237-B113] Zhong Y, et al RBFOX1 and working memory: from genome to transcriptome revealed posttranscriptional mechanism separate from attention-deficit/hyperactivity disorder. Biol Psychiatry Glob Open Sci. 2023:3:1042–1052. 10.1016/j.bpsgos.2022.08.006. PMC10593897 37881587

