# Supplementary material for: Lateralized Gene Splicing in the Brain is Associated With Lateralized Feeding Behavior in an Asymmetrical Scale-eating Cichlid Fish
Source: Genome Biol Evol. 2026 Oct 8;18(10):evag237. doi: 10.1093/gbe/evag237 (PMC13646608; doi:10.1093/gbe/evag237)
Supplement: Supplementary file 1 [file evag237_supplementary_data.zip › Final_Sup materials.GBE.NoRedMarked.docx]

**Supplementary materials**

**Lateralized gene splicing in the brain is associated with lateralized feeding behavior in an asymmetrical scale-eating cichlid fish**

**Authors:**

Xiaomeng Tian^1^, Axel Meyer^1,2,3^*

**Affiliations:**

^1^Department of Biology, University of Konstanz, Konstanz, Germany

^2^Museum of Comparative Zoology, Harvard University, Cambridge, MA, USA

^3^CAS Key Laboratory of Tropical Marine Bio-Resources and Ecology, South China Sea Institute of Oceanology, Chinese Academy of Sciences, Guangzhou 510301, China

*Corresponding authors. E-mail: [axel.meyer@uni-konstanz.de](mailto:axel.meyer@uni-konstanz.de) (A.M.).

**This PDF file includes:**

- Figures S1 to S10
- Tables S1 to S4
- Legends for Tables S5 to S7

**Other Supplementary Materials for this manuscript include the following:**

Table S5 to S7

**Fig. S1** Proportions of different types of alternative splicing events in brain regions collected under two states. **a**. Under resting state. **b**. Under predatory state. The five major splicing patterns are shown: alternative 3' splice sites (A3SS), alternative 5’ splice sites (A5SS), mutually exclusive exons (MXE), retained intron (RI) and skipped exon (SE).

**Fig. S2** Venn diagram of alternative spliced genes between dominant and non-dominant hemispheres across three brain regions under resting state conditions.

**Fig. S3** Venn diagram of alternative spliced genes between dominant and non-dominant hemispheres across three brain regions under predatory state conditions.

**Fig. S4** Alternative splicing patterns of *RIMS2* between dominant and non-dominant sides under resting (**a**) and predatory (**b**) states.

**Fig. S5** Structural changes in the DUF3694 domain of *KIF1A* after exon skipping. (**a**) Predicted protein structure of the isoform containing the exon of interest, with the DUF3694 domain highlighted in red and the exon of interest highlighted in yellow. (**b**) Predicted protein structure of the isoform after the exon of interest is skipped, with the position of the skipped exon highlighted in yellow. The white arrows indicate the two β-sheet strands affected by exon skipping.

**Fig. S6** *RIMS2* protein structure prediction and localization of the alternatively spliced exon. (**a**) Predicted structure of the exon-included isoform, with the exon of interest highlighted in red within predicted disordered loop regions. (**b**) Predicted structure of the exon-skipped isoform, with the skipped exon position highlighted in red. White arrows indicate the exon location.

**Fig. S7** *BPTF* protein structure prediction and localization of the alternatively spliced exon. (**a**) Predicted structure of the exon-included isoform, with the exon of interest highlighted in red within predicted disordered loop regions. (**b**) Predicted structure of the exon-skipped isoform, with the skipped exon position highlighted in red. White arrows indicate the exon location.

**Fig. S8** Top 30 enriched pathways of hemispheric alternatively spliced genes across three brain regions in the resting state: (**a**) Telencephalon, (**b**) Hindbrain, and (**c**) Optic tectum. Pathways closely related to the nervous system are highlighted in green, and pathways associated with microtubule functions are shown in orange.

**Fig. S9** Top 30 enriched pathways of hemispheric alternatively spliced genes across three brain regions in the predatory state: (**a**) Telencephalon, (**b**) Hypothalamus, and (**c**) Optic tectum. Pathways closely related to the nervous system are highlighted in green, and pathways associated with microtubule functions are shown in orange.

**Fig. S10** Enriched GO terms associated with alternative spliced genes shared by two brain regions under the predatory state conditions. GO terms related to nervous system or microtubule are highlighted in bold.

**Table S1** Top ten candidate genes showing the most significant splicing differences in exon inclusion levels between hemispheres across paired brain regions under the resting state.

|  | **Gene** | **Chr** | **Start** | **End** | **Strand** | **IncLevel** | **P value** | **Full name** |
| --- | --- | --- | --- | --- | --- | --- | --- | --- |
| Optic tectum | SLC35G1 | 8 | 14505970 | 14511384 | + | -0.319 | 4.06E-06 | solute carrier family 35 member G1 |
|  | KDM4C | 6 | 17511844 | 17540273 | + | -0.298 | 3.59E-07 | lysine demethylase 4C |
|  | RPS25 | 14 | 23921529 | 23923779 | + | -0.29 | 1.47E-06 | ribosomal protein S25 |
|  | MARCH1 | 6 | 26040239 | 26054434 | - | 0.269 | 7.82E-07 | membrane-associated ring finger (C3HC4) 1 |
|  | TOM1L2 | 4 | 16249557 | 16268847 | + | -0.263 | 3.00E-04 | target of myb1 like 2 membrane trafficking protein |
|  | RBFOX1 | 4 | 31844151 | 31988306 | + | -0.218 | 6.89E-06 | RNA binding fox-1 homolog 1 |
|  | FCHO2 | 7 | 50562180 | 50608407 | - | -0.217 | 5.60E-03 | FCH and mu domain containing endocytic adaptor 2 |
|  | RAP1GDS1 | 3 | 51753396 | 51783732 | + | -0.215 | 3.60E-03 | Rap1 GTPase-GDP dissociation stimulator 1 |
|  | MPP3 | 4 | 6943621 | 6966741 | - | -0.21 | 3.51E-04 | MAGUK p55 scaffold protein 3 |
|  | COMMD9 | 7 | 25601876 | 25607293 | - | 0.177 | 1.28E-02 | COMM domain containing 9 |
| Telencephalon | IFT43 | 19 | 26849449 | 26863799 | - | 0.365 | 2.51E-04 | intraflagellar transport 43 |
|  | MTUS1 | 2 | 3972202 | 3992233 | - | -0.26 | 9.23E-03 | microtubule associated scaffold protein 1 |
|  | CCDC85CB | 15 | 3072562 | 3117118 | + | -0.251 | 7.75E-04 | coiled-coil domain containing 85C, b |
|  | NUDT13 | 8 | 1043247 | 1046530 | - | 0.248 | 2.41E-03 | nudix hydrolase 13 |
|  | ASPH | 18 | 447782 | 453419 | - | -0.244 | 1.60E-03 | aspartate beta-hydroxylase |
|  | GPAA1 | 18 | 18482495 | 18486035 | - | 0.205 | 1.57E-04 | glycosylphosphatidylinositol anchor attachment 1 |
|  | TANGO2 | 12 | 4106131 | 4124020 | - | -0.204 | 1.64E-02 | transport and golgi organization 2 homolog |
|  | CAMK2G | 13 | 23035413 | 23113206 | - | 0.191 | 4.75E-03 | calcium/calmodulin dependent protein kinase II gamma |
|  | KLHL26 | 15 | 32650663 | 32655119 | - | -0.19 | 1.13E-02 | kelch like family member 26 |
|  | ADGRL2 | 15 | 37006966 | 37075615 | + | 0.189 | 1.89E-02 | adhesion G protein-coupled receptor L2 |
| Hindbrain | Unknown | 7 | 41470269 | 41472900 | - | 0.296 | 2.31E-02 | Uncharacterized gene |
|  | UBB | 10 | 7796495 | 7824572 | - | 0.242 | 1.80E-03 | ubiquitin B |
|  | RAB11FIP5 | 19 | 28962681 | 28987142 | + | 0.239 | 9.24E-07 | RAB11 family interacting protein 5 |
|  | KIF2A | 12 | 17574095 | 17596393 | + | 0.211 | 1.23E-08 | kinesin family member 2A |
|  | CHERP | 17 | 23785756 | 23806505 | - | 0.187 | 2.96E-03 | calcium homeostasis endoplasmic reticulum protein |
|  | PBX3 | 12 | 11935331 | 11989264 | - | -0.175 | 6.19E-04 | PBX homeobox 3 |
|  | NAA50 | 9 | 17127689 | 17130149 | - | 0.172 | 5.11E-06 | N-alpha-acetyltransferase 50, NatE catalytic subunit |
|  | RYR3 | 19 | 27671235 | 27785661 | - | 0.156 | 1.52E-02 | ryanodine receptor 3 |
|  | ZNF865 | 11 | 22820886 | 22825577 | + | -0.149 | 1.08E-04 | zinc finger protein 865 |
|  | MBP | 22 | 26027111 | 26033790 | - | 0.133 | 4.71E-02 | myelin basic protein |

**Table S2** Candidate genes exhibiting significant correlations between hemispheric exon inclusion levels in the hypothalamus and individuals' lateralized behavior. Pearson correlation coefficient (r) and corresponding *P*-value are indicated.

| **Gene** | **Chr** | **Start** | **End** | **Strand** | **IncLevel** | **Correlation** | **P value** | **Full name** |
| --- | --- | --- | --- | --- | --- | --- | --- | --- |
| MPRIP | 8 | 25176939 | 25220808 | - | -0.778 | 0.89 | 3.42E-03 | myosin phosphatase Rho interacting protein |
| CTAGE5 | 19 | 12347656 | 12363536 | + | 0.374 | -0.91 | 1.45E-03 | CTAGE family, member 5 |
| PHC1 | 22 | 30617306 | 30630402 | + | -0.36 | 0.76 | 2.79E-02 | polyhomeotic homolog 1 |
| BPTF | 4 | 25391846 | 25418300 | + | -0.35 | 0.83 | 1.10E-02 | bromodomain PHD finger transcription factor |
| MAGI2 | 20 | 26880775 | 26927074 | - | -0.323 | 0.77 | 2.42E-02 | membrane associated guanylate kinase, WW and PDZ domain containing 2 |
| MAGI2 | 20 | 26880775 | 26927074 | - | -0.317 | 0.77 | 2.39E-02 | membrane associated guanylate kinase, WW and PDZ domain containing 2 |
| BPTF | 4 | 25391846 | 25418300 | + | -0.286 | 0.85 | 7.15E-03 | bromodomain PHD finger transcription factor |
| ANXA6 | 2 | 7052632 | 7062756 | - | -0.263 | 0.74 | 3.59E-02 | annexin A6 |
| CABIN1 | 12 | 10603785 | 10641197 | + | -0.261 | 0.77 | 2.68E-02 | calcineurin binding protein 1 |
| KCNT1 | 12 | 29077703 | 29147897 | - | -0.254 | 0.86 | 6.69E-03 | potassium sodium-activated channel subfamily T member 1 |
| MAGI2 | 20 | 26880775 | 26927074 | - | -0.239 | 0.73 | 4.00E-02 | membrane associated guanylate kinase, WW and PDZ domain containing 2 |
| CDC14B | 12 | 24353911 | 24364577 | + | 0.231 | -0.78 | 2.34E-02 | cell division cycle 14B |
| RASGRF2 | 7 | 53696002 | 53744992 | + | -0.228 | 0.72 | 4.26E-02 | Ras protein specific guanine nucleotide releasing factor 2 |
| PLEKHA1 | 13 | 21331829 | 21343967 | - | -0.221 | 0.78 | 2.27E-02 | pleckstrin homology domain containing A1 |
| GRID1 | 13 | 20923784 | 21183387 | - | -0.197 | 0.74 | 3.57E-02 | glutamate ionotropic receptor delta type subunit 1 |
| HEXD | 8 | 22160307 | 22164635 | - | 0.196 | -0.73 | 4.02E-02 | hexosaminidase D |
| CTAGE5 | 19 | 12347656 | 12363536 | + | 0.186 | -0.91 | 1.84E-03 | CTAGE family, member 5 |
| GEMIN8 | 23 | 1194827 | 1195757 | + | 0.17 | -0.82 | 1.25E-02 | gem nuclear organelle associated protein 8 |
| MED20 | 5 | 18365030 | 18367425 | - | 0.165 | -0.75 | 3.35E-02 | mediator complex subunit 20 |
| STPG2 | 12 | 20252533 | 20306641 | + | -0.155 | 0.75 | 3.34E-02 | sperm tail PG-rich repeat containing 2 |
| PRSS12 | 6 | 20360085 | 20379522 | - | 0.137 | -0.77 | 2.50E-02 | serine protease 12 |
| MARK3 | 19 | 6705834 | 6751862 | - | -0.137 | 0.73 | 3.78E-02 | microtubule affinity regulating kinase 3 |
| NHS | 23 | 24357590 | 24376226 | - | -0.131 | 0.78 | 2.12E-02 | NHS actin remodeling regulator |
| CAMK2D | 3 | 52585830 | 52683305 | - | 0.129 | -0.71 | 4.67E-02 | calcium/calmodulin dependent protein kinase II delta |
| FN1 | 1 | 26438794 | 26456409 | + | 0.115 | -0.71 | 4.73E-02 | fibronectin 1 |
| PLEKHA5 | 7 | 15524915 | 15607703 | + | -0.106 | 0.77 | 2.42E-02 | pleckstrin homology domain containing A5 |
| MARK3 | 19 | 6705834 | 6751862 | - | -0.105 | 0.83 | 9.98E-03 | microtubule affinity regulating kinase 3 |
| FRMPD2 | 13 | 14688931 | 14710695 | + | -0.104 | 0.75 | 3.26E-02 | FERM and PDZ domain containing 2 |
| MARK3 | 19 | 6705834 | 6751862 | - | -0.102 | 0.80 | 1.61E-02 | microtubule affinity regulating kinase 3 |
| ANK1 | 7 | 37982824 | 38056674 | - | -0.1 | 0.87 | 5.22E-03 | ankyrin 1 |
| TAF1 | 10 | 11680124 | 11694204 | + | -0.099 | 0.77 | 2.66E-02 | TATA-box binding protein associated factor 1 |
| SOCS7 | 4 | 595982 | 602328 | - | -0.097 | 0.80 | 1.76E-02 | suppressor of cytokine signaling 7 |
| MBD3 | 23 | 31538188 | 31545499 | - | -0.096 | 0.83 | 1.08E-02 | methyl-CpG binding domain protein 3 |
| DNM1L | 12 | 14057228 | 14066728 | - | 0.094 | -0.78 | 2.22E-02 | dynamin 1 like |
| DIP2A | 16 | 13356333 | 13442203 | - | 0.091 | -0.72 | 4.47E-02 | disco interacting protein 2 homolog A |
| NUDT13 | 8 | 1043247 | 1046530 | - | -0.084 | 0.73 | 4.02E-02 | nudix hydrolase 13 |
| XKR5 | 1 | 14781876 | 14792857 | + | -0.081 | 0.81 | 1.58E-02 | XK related 5 |
| CHST1 | 7 | 25054091 | 25067864 | - | 0.074 | -0.72 | 4.44E-02 | carbohydrate sulfotransferase 1 |
| MARK3 | 19 | 6705834 | 6751862 | - | -0.07 | 0.84 | 8.72E-03 | microtubule affinity regulating kinase 3 |
| NADSYN1 | 1 | 1311455 | 1320146 | - | 0.063 | -0.78 | 2.29E-02 | NAD synthetase 1 |
| Unknown | 18 | 265876 | 287065 | + | -0.06 | 0.71 | 4.75E-02 | Uncharacterized gene |
| ANK1 | 7 | 37982824 | 38056674 | - | 0.057 | -0.78 | 2.30E-02 | ankyrin 1 |
| DLG3 | 2 | 15550555 | 15605442 | - | -0.057 | 0.90 | 2.38E-03 | discs large MAGUK scaffold protein 3 |
| TAFA1 | 5 | 10132217 | 10250131 | - | -0.049 | 0.93 | 7.18E-04 | TAFA chemokine like family member 1 |
| GRAP | 4 | 20327738 | 20361435 | - | -0.024 | 0.87 | 5.27E-03 | GRB2 related adaptor protein |
| FABP7 | 15 | 21172803 | 21173980 | - | 0.016 | -0.78 | 2.10E-02 | fatty acid binding protein 7 |

**Table S3** Candidate genes exhibiting significant correlations between hemispheric exon inclusion levels in the optic tectum and individuals' lateralized behavior. Pearson correlation coefficient (r) and corresponding *P*-value are indicated.

| **Gene** | **Chr** | **Start** | **End** | **Strand** | **IncLevel** | **Correlation** | **P value** | **Full name** |
| --- | --- | --- | --- | --- | --- | --- | --- | --- |
| POLR2B | 2 | 70064 | 108017 | + | 0.48 | -0.76 | 4.63E-02 | RNA polymerase II subunit B |
| ARHGAP17 | 4 | 16013831 | 16037804 | + | -0.467 | 0.77 | 4.40E-02 | Rho GTPase activating protein 17 |
| EIF4G3 | 5 | 28929782 | 28997480 | + | 0.453 | -0.86 | 1.34E-02 | eukaryotic translation initiation factor 4 gamma 3 |
| KCNMA1 | 8 | 12161884 | 12254193 | + | 0.442 | -0.80 | 2.95E-02 | potassium calcium-activated channel subfamily M alpha 1 |
| IDH3G | 20 | 31251871 | 31257882 | - | -0.436 | 0.89 | 7.71E-03 | isocitrate dehydrogenase (NAD(+)) 3 non-catalytic subunit gamma |
| TRDMT1 | 9 | 4175076 | 4190454 | - | -0.428 | 0.89 | 7.06E-03 | tRNA aspartic acid methyltransferase 1 |
| GABRB3 | 23 | 30758172 | 30802586 | - | -0.366 | 0.91 | 4.09E-03 | gamma-aminobutyric acid type A receptor subunit beta3 |
| RASAL2 | 18 | 32719773 | 32785254 | - | -0.363 | 0.82 | 2.44E-02 | RAS protein activator like 2 |
| cntn1 | 17 | 7775242 | 7783436 | + | 0.362 | -0.89 | 7.34E-03 | contactin 1 |
| PRADC1 | 12 | 26482586 | 26486530 | - | -0.343 | 0.79 | 3.53E-02 | protease associated domain containing 1 |
| CELF2 | 17 | 18518858 | 18752894 | + | 0.342 | -0.84 | 1.69E-02 | CUGBP Elav-like family member 2 |
| THOP1 | 7 | 51194235 | 51200950 | + | 0.309 | -0.83 | 2.14E-02 | thimet oligopeptidase 1 |
| FLNA | 17 | 22796967 | 22852897 | + | -0.282 | 0.93 | 2.59E-03 | filamin A |
| DLGAP4 | 20 | 25223181 | 25247356 | + | -0.263 | 0.92 | 3.68E-03 | DLG associated protein 4 |
| CNNM2 | 6 | 8966093 | 9015350 | - | -0.256 | 0.79 | 3.40E-02 | cyclin and CBS domain divalent metal cation transport mediator 2 |
| SH3BP5L | 22 | 332121 | 348818 | - | -0.246 | 0.86 | 1.24E-02 | SH3 binding domain protein 5 like |
| BDNF | 1 | 22658082 | 22671570 | + | -0.233 | 0.88 | 9.81E-03 | brain derived neurotrophic factor |
| SLAIN2 | 2 | 8436537 | 8445443 | - | -0.225 | 0.80 | 3.25E-02 | SLAIN motif family member 2 |
| ADGRL2 | 15 | 37006966 | 37075615 | + | 0.225 | -0.80 | 2.99E-02 | adhesion G protein-coupled receptor L2 |
| apopt1 | 19 | 6638149 | 6643813 | - | -0.216 | 0.77 | 4.45E-02 | apoptogenic 1, mitochondrial |
| TTLL5 | 19 | 26877529 | 26943145 | - | -0.211 | 0.92 | 3.23E-03 | tubulin tyrosine ligase like 5 |
| FLNA | 17 | 22796967 | 22852897 | + | -0.208 | 0.91 | 4.85E-03 | filamin A |
| ALDH1L2 | 17 | 36393647 | 36410626 | + | 0.206 | -0.90 | 5.55E-03 | aldehyde dehydrogenase 1 family member L2 |
| PLCG1 | 17 | 25222547 | 25263064 | + | -0.187 | 0.84 | 1.76E-02 | phospholipase C gamma 1 |
| SNX4 | 16 | 13064137 | 13070488 | + | -0.183 | 0.83 | 2.14E-02 | sorting nexin 4 |
| ATP1B2 | 3 | 1982726 | 1992796 | + | 0.161 | -0.90 | 5.33E-03 | ATPase Na+/K+ transporting subunit beta 2 |
| F9 | 10 | 19959768 | 19980413 | + | -0.159 | 0.88 | 8.36E-03 | coagulation factor IX |
| RRN3 | 4 | 31248255 | 31275506 | - | 0.157 | -0.82 | 2.54E-02 | RRN3 homolog, RNA polymerase I transcription factor |
| ZMYND12 | 20 | 6065303 | 6071934 | + | -0.155 | 0.78 | 3.98E-02 | zinc finger MYND-type containing 12 |
| FERMT2 | 19 | 20603604 | 20640684 | + | 0.151 | -0.94 | 1.72E-03 | FERM domain containing kindlin 2 |
| NLGN1 | 23 | 26975373 | 27215193 | + | -0.147 | 0.82 | 2.45E-02 | neuroligin 1 |
| EPHB2 | 20 | 18466815 | 18524351 | + | -0.138 | 0.84 | 1.84E-02 | EPH receptor B2 |
| CISH | 5 | 21911815 | 21913269 | + | -0.137 | 0.80 | 2.97E-02 | cytokine inducible SH2 containing protein |
| GRIN1 | 7 | 33718495 | 33752779 | - | -0.126 | 0.79 | 3.56E-02 | glutamate ionotropic receptor NMDA type subunit 1 |
| CLIP1 | 12 | 19765170 | 19787770 | + | -0.124 | 0.92 | 3.77E-03 | CAP-Gly domain containing linker protein 1 |
| SLC24A4 | 15 | 15031150 | 15062517 | - | -0.119 | 0.83 | 2.06E-02 | solute carrier family 24 member 4 |
| NSMCE4A | 13 | 11348597 | 11354500 | + | -0.112 | 0.80 | 3.22E-02 | NSE4 homolog A, SMC5-SMC6 complex component |
| GRIN1 | 7 | 33718495 | 33752779 | - | 0.111 | -0.82 | 2.28E-02 | glutamate ionotropic receptor NMDA type subunit 1 |
| DGKI | 17 | 17237628 | 17326873 | + | -0.11 | 0.84 | 1.89E-02 | diacylglycerol kinase iota |
| PPP6R2 | 7 | 10041632 | 10054072 | + | 0.108 | -0.86 | 1.40E-02 | protein phosphatase 6 regulatory subunit 2 |
| PHLDB2 | 9 | 12290928 | 12326774 | + | 0.104 | -0.93 | 2.59E-03 | pleckstrin homology like domain family B member 2 |
| EEIG2 | 17 | 29985545 | 30004383 | - | 0.1 | -0.80 | 3.22E-02 | EEIG family member 2 |
| RBM5 | 5 | 17690509 | 17698253 | - | 0.092 | -0.85 | 1.62E-02 | RNA binding motif protein 5 |
| CRTC2 | 11 | 36766078 | 36791606 | - | -0.083 | 0.80 | 3.21E-02 | CREB regulated transcription coactivator 2 |
| PCLO | 17 | 12190001 | 12244922 | + | -0.081 | 0.81 | 2.73E-02 | piccolo presynaptic cytomatrix protein |
| GLTSCR1 | 14 | 6668546 | 6677798 | + | -0.077 | 0.87 | 1.04E-02 | BRD4 interacting chromatin remodeling complex associated protein |
| ABCA12 | 13 | 24977894 | 25008511 | + | -0.064 | 0.80 | 3.07E-02 | ATP binding cassette subfamily A member 12 |
| SEC24C | 12 | 30911373 | 30922031 | - | -0.054 | 0.82 | 2.44E-02 | SEC24 homolog C, COPII coat complex component |
| ARHGAP17 | 8 | 20541633 | 20579827 | - | 0.053 | -0.82 | 2.47E-02 | Rho GTPase activating protein 17 |
| SRP19 | 12 | 35983849 | 35987147 | + | -0.049 | 0.80 | 3.26E-02 | signal recognition particle 19 |
| FAM131B | 22 | 30707374 | 30783716 | - | -0.046 | 0.87 | 1.15E-02 | family with sequence similarity 131 member B |
| RIMS1 | 13 | 4263190 | 4372956 | - | 0.034 | -0.95 | 1.31E-03 | regulating synaptic membrane exocytosis 1 |
| KIFAP3 | 18 | 5501602 | 5517810 | - | -0.029 | 0.94 | 1.47E-03 | kinesin associated protein 3 |

**Table S4** Candidate genes exhibiting significant correlations between hemispheric exon inclusion levels in the telencephalon and individuals' lateralized behavior. Pearson correlation coefficient (r) and corresponding *P*-value are indicated.

| **Gene** | **Chr** | **Start** | **End** | **Strand** | **IncLevel** | **Correlation** | | **P value** | **Full name** |
| --- | --- | --- | --- | --- | --- | --- | --- | --- | --- |
| ZZEF1 | 10 | 1083743 | 1111919 | - | -0.51 | 0.82 | 1.24E-02 | | zinc finger ZZ-type and EF-hand domain containing 1 |
| NDUFV3 | 16 | 32040136 | 32049260 | + | -0.483 | 0.76 | 2.72E-02 | | NADH:ubiquinone oxidoreductase subunit V3 |
| PPCDC | 7 | 12850408 | 12858626 | - | -0.362 | 0.77 | 2.57E-02 | | phosphopantothenoylcysteine decarboxylase |
| PPP1R10 | 22 | 3451798 | 3460410 | - | -0.347 | 0.82 | 1.28E-02 | | protein phosphatase 1 regulatory subunit 10 |
| MEF2D | 22 | 20813917 | 20833817 | + | 0.344 | -0.85 | 7.06E-03 | | myocyte enhancer factor 2D |
| RIMS2 | 22 | 9939288 | 9993690 | - | 0.342 | -0.92 | 1.37E-03 | | regulating synaptic membrane exocytosis 2 |
| PQLC3 | 19 | 12880946 | 12890016 | - | -0.325 | 0.73 | 3.79E-02 | | PQ loop repeat containing 3 |
| YLPM1 | 15 | 9340952 | 9366232 | + | -0.287 | 0.86 | 5.59E-03 | | YLP motif containing 1 |
| KIFC3 | 1 | 2613097 | 2640284 | + | 0.285 | -0.91 | 1.60E-03 | | kinesin family member C3 |
| ZNF160 | 9 | 29627441 | 29636499 | - | -0.284 | 0.77 | 2.68E-02 | | zinc finger protein 160 |
| DAB1 | 23 | 35750248 | 35798310 | - | -0.277 | 0.83 | 1.17E-02 | | DAB adaptor protein 1 |
| TRIO | 11 | 11756219 | 11865591 | + | -0.267 | 0.75 | 3.29E-02 | | trio Rho guanine nucleotide exchange factor |
| GTF2A2 | 1 | 2437181 | 2439258 | - | -0.263 | 0.72 | 4.47E-02 | | general transcription factor IIA subunit 2 |
| PAXX | 12 | 8861509 | 8863793 | - | -0.25 | 0.81 | 1.54E-02 | | AXX non-homologous end joining factor |
| KIF21B | 5 | 13886219 | 13946097 | + | -0.249 | 0.84 | 8.75E-03 | | kinesin family member 21B |
| SYTL1 | 22 | 15999560 | 16007711 | - | 0.247 | -0.83 | 1.10E-02 | | synaptotagmin like 1 |
| GNS | 7 | 55313756 | 55324500 | - | -0.241 | 0.78 | 2.11E-02 | | glucosamine (N-acetyl)-6-sulfatase |
| SASH1 | 15 | 10898651 | 11060313 | - | -0.232 | 0.83 | 1.11E-02 | | SAM and SH3 domain containing 1 |
| RFX2 | 17 | 1867897 | 1887621 | + | 0.223 | -0.71 | 4.69E-02 | | regulatory factor X2 |
| TMEM234 | 19 | 22824068 | 22828522 | + | -0.222 | 0.80 | 1.60E-02 | | transmembrane protein 234 |
| DCAF6 | 16 | 20170671 | 20201869 | - | 0.193 | -0.74 | 3.76E-02 | | DDB1 and CUL4 associated factor 6 |
| ATXN2 | 12 | 24662283 | 24681669 | + | -0.191 | 0.88 | 4.39E-03 | | ataxin 2 |
| FAM149B1 | 13 | 20710509 | 20720226 | + | -0.185 | 0.84 | 9.81E-03 | | family with sequence similarity 149 member B1 |
| nos1ap | 18 | 10171889 | 10191509 | - | 0.175 | -0.84 | 8.37E-03 | | nitric oxide synthase 1 adaptor protein |
| TVP23A | 7 | 6981789 | 6984450 | - | -0.169 | 0.84 | 9.27E-03 | | trans-golgi network vesicle protein 23 homolog A |
| atxn7l3 | 4 | 6480978 | 6485609 | + | -0.168 | 0.87 | 4.63E-03 | | ataxin 7 like 3 |
| YLPM1 | 15 | 9340952 | 9366232 | + | -0.165 | 0.86 | 6.26E-03 | | YLP motif containing 1 |
| KIFC3 | 1 | 2613097 | 2640284 | + | 0.157 | -0.81 | 1.55E-02 | | kinesin family member C3 |
| KIF1A | 23 | 29661638 | 29706274 | + | 0.154 | -0.87 | 5.34E-03 | | kinesin family member 1A |
| DVL1 | 20 | 26542931 | 26564634 | - | -0.149 | 0.71 | 4.65E-02 | | dishevelled segment polarity protein 1 |
| HDX | 2 | 4297460 | 4302260 | - | -0.138 | 0.78 | 2.38E-02 | | highly divergent homeobox |
| PCSK9 | 23 | 35532636 | 35540704 | + | 0.126 | -0.79 | 2.03E-02 | | proprotein convertase subtilisin/kexin type 9 |
| SMG1 | 4 | 31421508 | 31477836 | - | 0.122 | -0.87 | 4.50E-03 | | SMG1 nonsense mediated mRNA decay associated PI3K related kinase |
| FAM189B | 11 | 21854814 | 21863889 | + | -0.121 | 0.86 | 6.07E-03 | | family with sequence similarity 189 member B |
| MYSM1 | 23 | 36024809 | 36030313 | - | -0.095 | 0.87 | 4.47E-03 | | myb-like, SWIRM and MPN domains 1 |
| TMEM237 | 23 | 17107588 | 17114478 | + | 0.09 | -0.72 | 4.52E-02 | | transmembrane protein 237 |
| SLC24A3 | 2 | 14370403 | 14385626 | + | -0.079 | 0.72 | 4.36E-02 | | solute carrier family 24 member 3 |
| LIPA | 8 | 938847 | 944519 | + | 0.076 | -0.72 | 4.28E-02 | | lipase A, lysosomal acid type |
| CTBP2 | 8 | 10911732 | 10963266 | + | 0.075 | -0.79 | 1.93E-02 | | C-terminal binding protein 2 |
| HECW2 | 23 | 2022933 | 2085086 | + | -0.056 | 0.90 | 2.15E-03 | | HECT, C2 and WW domain containing E3 ubiquitin protein ligase 2 |
| MTUS2 | 14 | 8385403 | 8403827 | - | 0.053 | -0.76 | 2.78E-02 | | microtubule associated scaffold protein 2 |
| MTUS2 | 14 | 8385403 | 8403827 | - | 0.052 | -0.87 | 5.10E-03 | | microtubule associated scaffold protein 2 |
| BTBD6 | 19 | 6530861 | 6533922 | + | 0.045 | -0.74 | 3.57E-02 | | BTB domain containing 6 |
| ADAM17 | 19 | 24771686 | 24786756 | + | 0.029 | -0.75 | 3.23E-02 | | ADAM metallopeptidase domain 17 |

**Table S5** (separate file). Candidate genes exhibiting significant correlations between expression levels in the hypothalamus and individuals' lateralized behavior. Pearson correlation coefficient (r) and corresponding P-value are indicated.

**Table S6** (separate file). Candidate genes exhibiting significant correlations between expression levels in the optic tectum and individuals' lateralized behavior. Pearson correlation coefficient (r) and corresponding P-value are indicated.

**Table S7** (separate file). Candidate genes exhibiting significant correlations between expression levels in the telencephalon and individuals' lateralized behavior. Pearson correlation coefficient (r) and corresponding P-value are indicated.
